# Genetic Screen in *Drosophila* Larvae Links *ird1* Function to Toll Signaling in the Fat Body and Hemocyte Motility

**DOI:** 10.1371/journal.pone.0159473

**Published:** 2016-07-28

**Authors:** Martin R. Schmid, Ines Anderl, Hoa T. M. Vo, Susanna Valanne, Hairu Yang, Jesper Kronhamn, Mika Rämet, Tor Erik Rusten, Dan Hultmark

**Affiliations:** 1 Department of Molecular Biology, Umeå University, Umeå, Sweden; 2 BioMediTech, University of Tampere, Tampere, Finland; 3 PEDEGO Research Center, and Medical Research Center Oulu, University of Oulu and Oulu University Hospital, Oulu, Finland; 4 Department of Molecular Cell Biology, Oslo University Hospital, Centre for Cancer Biomedicine, University of Oslo, Oslo, Norway; University of Massachusetts Medical School, UNITED STATES

## Abstract

To understand how Toll signaling controls the activation of a cellular immune response in *Drosophila* blood cells (hemocytes), we carried out a genetic modifier screen, looking for deletions that suppress or enhance the mobilization of sessile hemocytes by the gain-of-function mutation *Toll*^*10b*^ (*Tl*^*10b*^). Here we describe the results from chromosome arm 3R, where five regions strongly suppressed this phenotype. We identified the specific genes *immune response deficient 1* (*ird1*), *headcase* (*hdc*) and possibly *Rab23* as suppressors, and we studied the role of *ird1* in more detail. An *ird1* null mutant and a mutant that truncates the N-terminal kinase domain of the encoded Ird1 protein affected the *Tl*^*10b*^ phenotype, unlike mutations that affect the C-terminal part of the protein. The *ird1* null mutant suppressed mobilization of sessile hemocytes, but enhanced other *Tl*^*10b*^ hemocyte phenotypes, like the formation of melanotic nodules and the increased number of circulating hemocytes. *ird1* mutants also had blood cell phenotypes on their own. They lacked crystal cells and showed aberrant formation of lamellocytes. *ird1* mutant plasmatocytes had a reduced ability to spread on an artificial substrate by forming protrusions, which may explain why they did not go into circulation in response to Toll signaling. The effect of the *ird1* mutation depended mainly on *ird1* expression in hemocytes, but *ird1*-dependent effects in other tissues may contribute. Specifically, the Toll receptor was translocated from the cell membrane to intracellular vesicles in the fat body of the *ird1* mutant, and Toll signaling was activated in that tissue, partially explaining the *Tl*^*10b*^-like phenotype. As *ird1* is otherwise known to control vesicular transport, we conclude that the vesicular transport system may be of particular importance during an immune response.

## Introduction

The core components of the Toll signaling pathway were initially identified in a classical genetic screen for mutations that affect *Drosophila* embryonic development, where Toll signaling was found to control dorsal-ventral polarity [1–3]. Later, the Toll pathway was also shown to activate a set of genes that code for antimicrobial peptides in *Drosophila* [4,5]. These peptides are effectors of the humoral arm of the phylogenetically highly conserved defense system of innate immunity (reviewed in [6,7]), serving as a first line of defense after infection in all higher organisms. The Toll pathway is named after the *Toll* gene, which encodes a transmembrane receptor. This receptor, Toll, interacts with the products of the *Myd88*, *pelle*, *tube* and *cactus* genes to activate two NF-κB-like transcription factors, encoded by the *dorsal* and *Dif* genes [8,9]. Together with a second NF-κB-like signaling pathway, the Imd pathway [5], the Toll pathway plays a major role in the induction of the humoral immune response in *Drosophila* (reviewed in [8,10,11]). Toll homologs were later identified in the mouse as well as in humans [12,13], where Toll-like receptors (TLRs) are crucial for activating and coordinating both innate and acquired immunity.

Genetic screens that target the immune response directly have later helped to identify additional genes that affect Toll and Imd signaling. One of them is the *immune response deficient 1* (*ird1*) gene, which was found by Wu *et al*. [14] in an ethyl methanesulfonate screen for mutations that suppress the Imd-dependent induction by bacteria of the antibacterial peptide Diptericin. In contrast to the impaired induction of Diptericin and several other Imd pathway-dependent antimicrobial peptides, homozygous *ird1* mutants showed constitutive expression of another antimicrobial peptide gene, *Drosomycin* [15], a target of both Toll and Imd signaling. This suggested that the Toll pathway is activated in *ird1* mutants. In support of this conclusion, Wu *et al*. also observed spontaneous formation of melanotic nodules in *ird1* null mutants [14]. This is a well-known phenotype for *Toll* gain-of-function mutants such as *Tl*^*10b*^ [16,17].

The *ird1* gene encodes a homolog of the yeast protein Vacuolar protein sorting 15 (Vps15) [15], a serine/threonine kinase with WD40 domains, which acts as a regulatory subunit of the class III phosphoinositide 3-kinase Vps34, also known in *Drosophila* as Pi3K59F. Together with Atg6 (Beclin 1) and Uvrag (Vps38) or Atg14 they form a PI 3-kinase complex that is involved in vesicle fusion events during phagocytosis, autophagy and endocytosis [18]. How a mutation in the PI 3-kinase complex affects the induction of antimicrobial peptide synthesis is unknown (See Ref. [19]).

While the humoral antimicrobial immune response is now well understood in *Drosophila* [9–11,20], less is known at the molecular level about the other arm of *Drosophila* innate immunity, the cellular immune defense [21,22]. This defense is mediated by blood cells, called hemocytes, which participate in the phagocytosis of microbes and the encapsulation of parasites [23]. Three classes of hemocytes are morphologically distinguishable in *Drosophila*: plasmatocytes, crystal cells and lamellocytes. The phagocytically active plasmatocytes constitute the major class, about 95% of the circulating hemocyte population in unchallenged larvae. There is also a large population of sessile plasmatocytes, many of which decorate the inside of the epidermis. The crystal cells contribute the remaining 5% and they are involved in the melanization of wounds and perhaps also of parasites [24]. Lamellocytes, the third class, are big flat cells that encapsulate parasites and foreign objects. They only appear in circulation during parasite infection or after other major challenges, when they can constitute up to 50% of the hemocytes. For instance, when a *Drosophila* larva is infected with eggs of a parasitoid wasp like *Leptopilina boulardi*, sessile plasmatocytes go into circulation and a subset of the plasmatocytes differentiate to become lamellocytes [25–28]. Larval hemocytes originate directly from a group of embryonic hemocytes, whereas many pupal and adult hemocytes are formed in a separate hematopoietic tissue, the so-called “lymph glands” that release their content of hemocytes at the larva-to-pupa metamorphosis [29]. A parasite infection can also lead to lamellocyte formation in the lymph glands, which then release their contents prematurely.

Several signaling pathways can cause release of sessile hemocytes and formation of lamellocytes [16,17,30], thus mimicking the response to parasite infection. This phenotype is for instance seen in the *Toll*^*10b*^ (*Tl*^*10b*^) mutant, in which the Toll pathway is constitutively activated. This effect is cell non-autonomous and mainly dependent on Toll activity in the fat body, not in hemocytes [31]. To identify genes that are involved in Toll-mediated hemocyte activation, we have now set up a genetic modifier screen for genes that affect the *Toll*^*10b*^ hemocyte phenotype. Here we report results for chromosome arm 3R, and we show that mutations in the *ird1* gene suppress normal hemocyte mobilization, but enhance many other aspects of hemocyte activation.

## Results and Discussion

### A genetic modifier screen

The phenotype of a dominant mutant, such as the gain-of function *Toll* mutant *Tl*^*10b*^ [32], can be used as a starting point to screen for mutations that modify this phenotype, either enhance or suppress it. We initiated such a modifier screen, to identify yet unknown components involved in *Toll*-dependent blood cell activation. In this way we expected to find genes involved in intercellular interactions between blood cells or between other tissues like the fat body and blood cells, as well as cell-autonomously acting genes. For this purpose we crossed *Tl*^*10b*^ flies with flies from the DrosDel collection of isogenized deletion mutants (“deficiencies”) [33], or with additional deficiencies from the Exelixis collection [34], in order to achieve higher coverage for genomic regions of particular interest. With a limited number of genetic crosses this approach makes it possible to screen the major part of the *Drosophila* genome for the effect of a 50% reduction of gene dosage.

As readout for hemocyte activation we chose to score the *Toll*-dependent mobilization of the segmental stripes of sessile hemocytes under the epidermis of undisturbed larvae. To visualize these cells *in vivo*, we introduced a *UAS-GFP* fluorescent construct, together with the hemocyte-specific driver *Hml*^*Δ*^*-GAL4*, into our *Tl*^*10b*^ stock (called *Hml>GFP*; *Tl*^*10b*^). Here we describe results for the right arm of the third chromosome, one fifth of the genome, which is covered to about 90% in our screen.

### Results of the deletion screen

**Fig 1:**
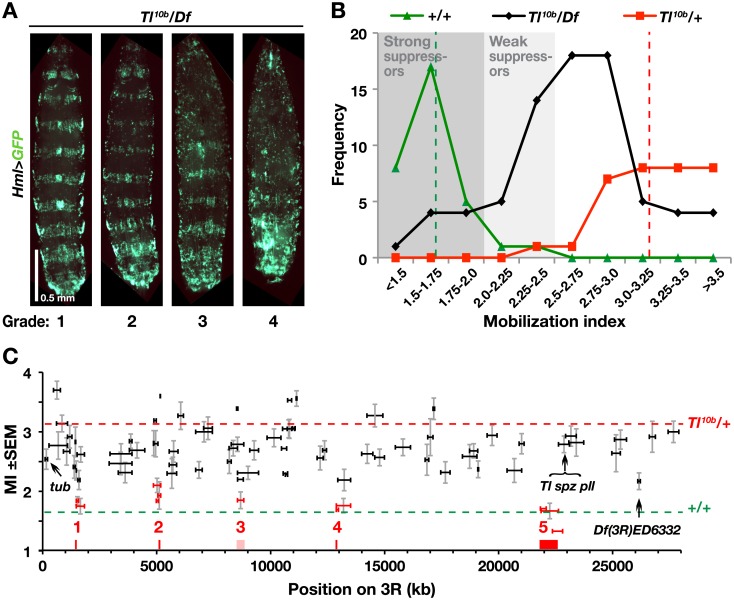
A genomic deletion screen on chromosome arm 3R identifies five candidate regions that suppress the sessile hemocyte disruption phenotype of *Tl*^*10b*^. **A.** Third instar larvae from *Tl*^*10b*^/deficiency (*Tl*^*10b*^/*Df*) crosses, representing the four phenotype grades, categorized according to the number of body segments with intact bands of sessile cells visualized with *Hml*^*Δ*^*-GAL4*-driven *UAS-GFP* (*Hml*>*GFP*). The larvae are oriented with the anterior end up. **B.** Frequency distribution of mobilization indices, calculated as the average grade for larvae of each genotype. Each point shows the number of *Tl*^*10b*^/*Df* crosses that gave mobilization indices in the indicated interval (black line), compared to the control *Tl*^*10b*^ (red line) and wild-type (green line) crosses. Dashed red and green lines indicate the average mobilization index of all *Tl*^*10b*^ (3.21, SD = 0.36) and wild-type (1.66, SD = 0.25) control crosses, respectively. Grey areas show our classification of the deficiencies as weak or strong suppressors. **C.** All tested deficiencies, plotted on the x-axis according to size and relative position on the right arm of the third chromosome, and on the y-axis according to mobilization index (MI, +/- standard error of mean, *n* = 16–22). Deficiencies categorized as strong suppressors are depicted in red. The red and green dashed lines indicate the average mobilization indices for the *Tl*^*10b*^ and control crosses respectively, the latter corresponding to full suppression of the *Tl*^*10b*^ phenotype.

We assayed the mobilization of activated hemocytes by scoring the disruption of the bands of sessile hemocytes on an arbitrary scale from 1 to 4 (Fig 1A), as described previously [31,35]. For each genotype we calculated a mobilization index (MI), defined as the average score of several individual larvae. Comparing the scores for all positive and negative control crosses during the course of this study, we found significantly higher (p<0.0001, chi-square test) mobilization indices for the *Tl*^*10b*^ crosses (mean MI = 3.21), compared to the wild-type controls (mean MI = 1.66), and there was little overlap between the scores of these cohorts (Fig 1B; S1 Table). The mobilization indices of all examined deficiency crosses form a broad distribution between these values. Of the 88 deficiencies tested, 13 overlapped with the wild-type phenotype and hence were categorized as strong suppressors. In addition, 17 deficiencies fell in the interval between the positive and negative controls and were classified as weak suppressors (Fig 1B and S1 Table). Due to the strong phenotype of the *Tl*^*10b*^ mutant in this genetic background, it was difficult to identify enhancers. However, given that *Tl*^*10b*^ is homozygous lethal [32], the ten deficiencies that failed to produce any offspring when crossed to *Hml>GFP*; *Tl*^*10b*^ could be regarded as potential enhancers (S1 Table).

All together, the strong suppressor deficiencies defined five minimal genomic regions of overlap (highlighted in red in Fig 1C), each harboring between 5 and 90 genes (Table 1). Notably, these regions did not include any of the classical Toll pathway genes of this chromosome arm: *tube*, *spätzle*, *pelle* or *Toll*. We concluded that a 50% reduced gene dosage of these genes was not sufficient to suppress the *Tl*^*10b*^ phenotype we investigated. Using a candidate gene approach, testing mutants and RNAi constructs of selected genes from these regions (for details, see S1 Text and S1 Fig), we identified two genes that affected the *Tl*^*10b*^ phenotype: *Rab23* in region 1 (S1A and S1B Fig) and *immune response deficient 1* (*ird1*) in region 2 (S1C and S1D Fig, see also further description below). Regions 3–5 were not further analyzed. Region 3 was problematic, as it overlapped with two larger non-suppressor deficiencies. Region 4 with 24 genes and region 5 with 90 genes were too large to be analyzed with our present phenotypic assays and they will require further mapping with additional deletions, which were not available at the time (Table 1). We did however investigate one weak suppressor deficiency *Df(3R)ED6332* with just four genes, and identified one of them, *headcase* (*hdc*), as a true suppressor (Fig 1C, S2 Fig). We will here focus on the role of *ird1* gene in Toll-dependent hemocyte activation.

**Table 1:** Suppressor regions.

Sup-pressor regions	Location on 3R (bp)	Genes in the region (tested genes in bold)					
**1**	1480524–1510301	*elm*					
		*MED27*					
		*CG2182*					
		*CR44093*					
		*CG1109*					
		*Xe7*					
		*Atg17*					
		***Rab23***					
**2**	5055517–5073219	***pum***					
		*CR45196*					
		***D1***					
		***ird1***					
		*CG8420*					
**3** ^**a**^	8545707–8821397	*CR44231*	*CG10126*	*CG14384*			
		*CG5724*	*CR44235*	*CG7381*			
		*CG5999*	*d-cup*	*CG7091*			
		*beat-Vc*	*CR33929*	*Paip2*			
		*CR44233*	*CG10909*	*CG31342*			
		*CR44232*	*beat-Vb*	*CG14383*			
		*CG31345*	*CR44236*	*yellow-f*			
		*beat-Va*	*grsm*	*yellow-f2*			
		*CR45587*	*Spc25*	*CG7488*			
		*CR44234*	*Cyp304a1*				
**4**	12879384–12974829	*Dad*	*CG17477*	*TwdlW*			
		*Ns1*	*CR44238*	*m-cup*			
		*mRpS11*	*CG31269*				
		*Keap1*	*CG31265*				
		*kuk*	*CG17475*				
		*GckIII*	*CG4053*				
		*cal1*	*CG31266*				
		*cher*	*CG31267*				
		*CG34278*	*CG5246*				
		*CG42779*	*CG5255*				
		*CG45079*	*CG5265*				
**5**	21832109–22624758	*E(spl)mα-BFM*	*Vps2*	*CG31323*	*CG5432*	*scrib*	*Tb*
		*Kaz-m1*	*Dak1*	*CG31086*	*CG6503*	*CR44951*	*TwdlQ*
		*CR45040*	*Sld5*	*CG6073*	*CG34291*	*TwdlM*	*amon*
		*E(spl)m2-BFM*	*CG14543*	*CG34130*	*CR31084*	*TwdlP*	*CG6425*
		*CR43643*	*CG42498*	*CG34129*	*CR43434*	*TwdlB*	*CG5484*
		*CR43644*	*CG14550*	*CR44320*	*tx*	*TwdlL*	*CG6420*
		*E(spl)m3-HLH*	*RpL34a*	*CG12290*	*Hex-t1*	*TwdlO*	*CG14244*
		*E(spl)m4-BFM*	*CG14544*	*CR42745*	*Hex-t2*	*TwdlK*	*CG14246*
		*E(spl)m5-HLH*	*CG14551*	*Ald*	*CG5447*	*TwdlJ*	*CG14245*
		*E(spl)m6-BFM*	*CR44950*	*CR44321*	*CG43117*	*TwdlN*	*CG6403*
		*E(spl)m7-HLH*	*dys*	*CG6154*	*Pdf*	*TwdlH*	*CG14247*
		*E(spl)m8-HLH*	*CR44319*	*CG33970*	*CG14237*	*TwdlR*	*CR45562*
		*gro*	*CG31324*	*CR42765*	*CG14238*	*TwdlS*	*CR45563*
		*Exo84*	*CG14556*	*CG6036*	*CG5455*	*TwdlD*	*CG42789*
		*CR45226*	*CG6142*	*ppk15*	*plum*	*beat-VII*	*CG42790*
**6** ^**b**^	26103647–26215013	***hdc***					
		*CG34300*					
		*Fer1HCH*					
		*Fer2LCH*					

^**a**^ Region covered by both suppressive and non-suppressive deletions

^**b**^ Weak suppressor region

### Mutations in the kinase domain of *ird1* suppress *Tl*^*10b*^-induced hemocyte mobilization, but enhance melanotic nodulation

**Fig 2:**
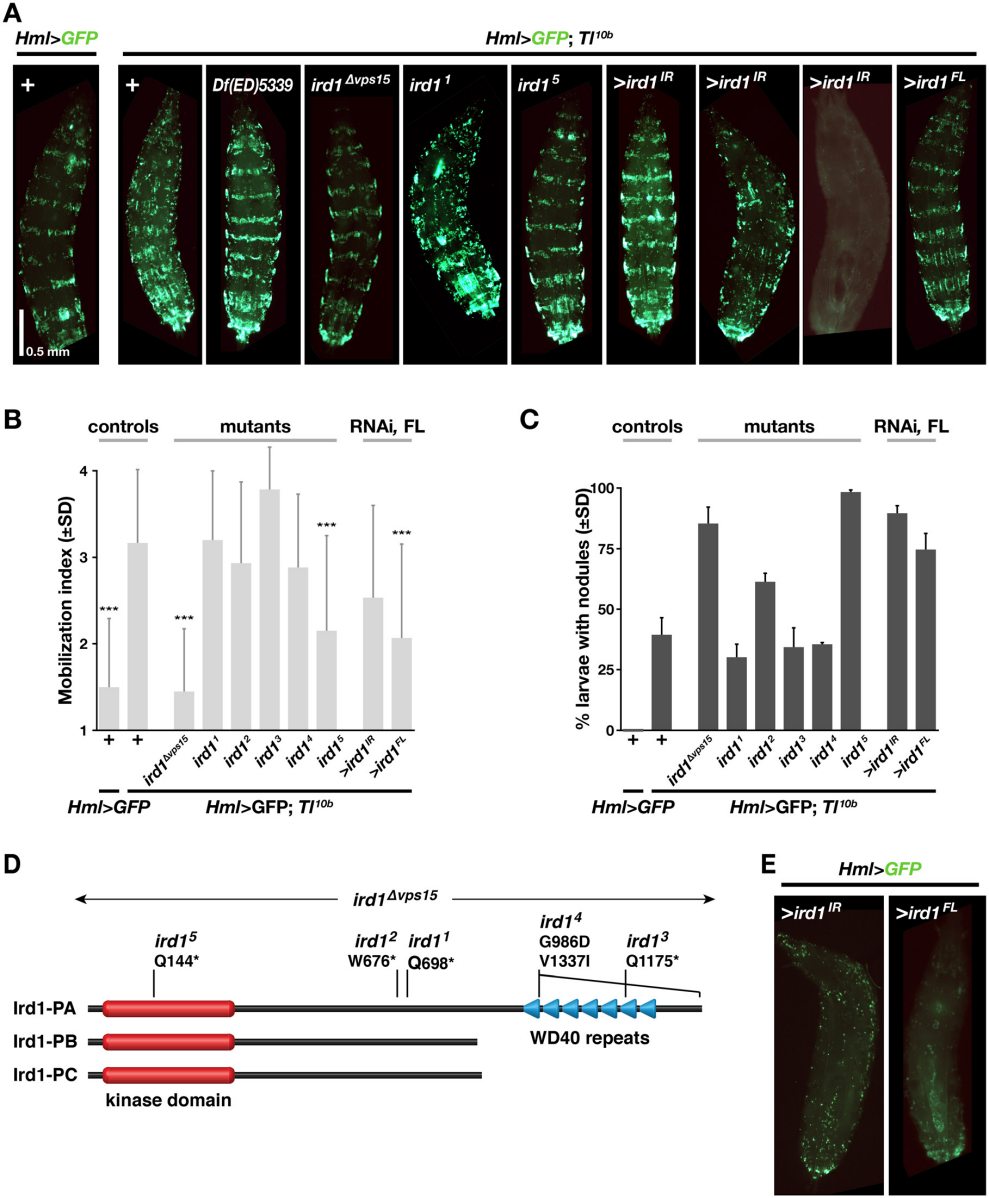
Modification of *Tl*^*10b*^ phenotype by *ird1* mutations, *ird1* RNAi and *ird1* overexpression. **A**. Sessile hemocyte patterns, as visualized with the *Hml*^*Δ*^*-GAL4* driven *UAS-GFP* reporter in control and *Tl*^*10b*^ larvae in combination with heterozygous *ird1* mutations, or after suppression or overexpression of *ird1* specifically in blood cells. **B**, Average mobilization index, pooled from three independent experiments, with 20 graded larvae in each experiments (+/- standard deviation, *n* = 60), and **C.** percentage of offspring with at least one melanotic nodule in three independent experiments, with 50 larvae in each experiment inspected for nodules (+/- standard deviation, *n* = 3). (***) Significant difference compared to the *Tl*^*10b*^ mutant control (*p*<0.0001), as estimated by pairwise comparisons using Kruskal-Wallis ANOVA test. Non-significant differences are not indicated. **D.** Schematic representation of the three Ird1 isoforms, and the mutants we have analyzed. **E.** Sessile hemocyte patterns in a *Toll* wild-type background, after suppression or overexpression of *ird1*.

To confirm the role of *ird1* in modifying the *Tl*^*10b*^ hemocyte phenotype, we tested the deletion mutant *ird1*^*Δvps15*^, created by Lindmo *et al*. [36], and found that even as heterozygote it is a strong suppressor of hemocyte mobilization when crossed to *Hml>GFP*; *Tl*^*10b*^ (Fig 2A and 2B). We also analyzed five point mutants, *ird1*^*1*^-*ird1*^*5*^, created by Wu *et al*. [14,15]. Except for *ird1*^*4*^, they are all nonsense mutants, predicted to encode truncated proteins (Fig 2D), and all five fail to complement the lethality of the original *ird1* mutant allele [14,15]. However we only could mimic the effect of the *ird1*^*Δvps15*^ deletion with the *ird1*^*5*^ mutant, which significantly suppressed the *Tl*^*10b*^ phenotype (Fig 2A and 2B). Notably, *ird1*^*5*^ is also the only allele that disrupts the kinase domain, whereas the other point mutations affect the C-terminal half of the Ird1 protein, disrupting or completely deleting the conserved WD40 repeats (Fig 2D). We conclude that the WD40 repeat region is mostly dispensable for the *Tl*^*10b*^ hemocyte phenotype we assayed. Of the three splice forms annotated for *ird1* in FlyBase [37], two encode truncated isoforms where the WD40 region is missing (Fig 2D). This suggests that such truncated Ird1 protein variants may also have a biological function *in vivo*, a function that would be insensitive to loss of the WD40 repeats.

Wu *et al*. [14,15] demonstrated that Toll signaling is constitutively activated in *ird1* homozygous mutants, activating both humoral and cellular immunity. We were therefore surprised to see that the kinase domain mutants *ird1*^*Δvps15*^ and *ird1*^*5*^ suppressed *Toll*-dependent hemocyte mobilization. On the other hand, the same mutant alleles and to a lesser extent *ird1*^*2*^, enhanced the *Tl*^*10b*^-induced formation of melanotic nodules (Fig 2C).

### Suppression of *ird1* expression in hemocytes is sufficient to modify the *Tl*^*10b*^ phenotype

We have previously shown that the effect of *Tl*^*10b*^ on hemocyte activation depends primarily on activated Toll signaling in the fat body, not in the hemocytes [31]. When we suppressed Toll signaling in hemocytes, the *Tl*^*10b*^ hemocyte phenotype was even enhanced. In order to understand the role of the *ird1* gene in this context, we examined in which tissue *ird1* exerts its effect on the *Tl*^*10b*^ phenotype. We first tested the role of *ird1* in hemocytes, by crossing an *ird1* RNAi transgene (*UAS-ird1*^*IR*^ [38]) into the *Hml>GFP*; *Tl*^*10b*^ stock. Like with the *ird1*^*Δvps15*^ and *ird1*^*5*^ mutants, this increased the percentage of larval offspring with nodules, compared to the *Tl*^*10b*^ mutant alone (Fig 2C, S3A Fig), although the average mobilization index of sessile hemocytes was unaffected (Fig 2B). However, in individual larvae of this genotype the sessile hemocytes showed an interesting range of phenotypes. While some larvae looked almost like wild-type with broad and regular bands of sessile blood cells, other larvae showed either a much disturbed pattern or a general reduction in *Hml*>*GFP* expression (Fig 2A shows three examples). Even in the absence of the *Tl*^*10b*^ mutation, the latter phenotype could be produced by blood cell-specific knockdown of *ird1* (Fig 2E).

**Fig 3:**
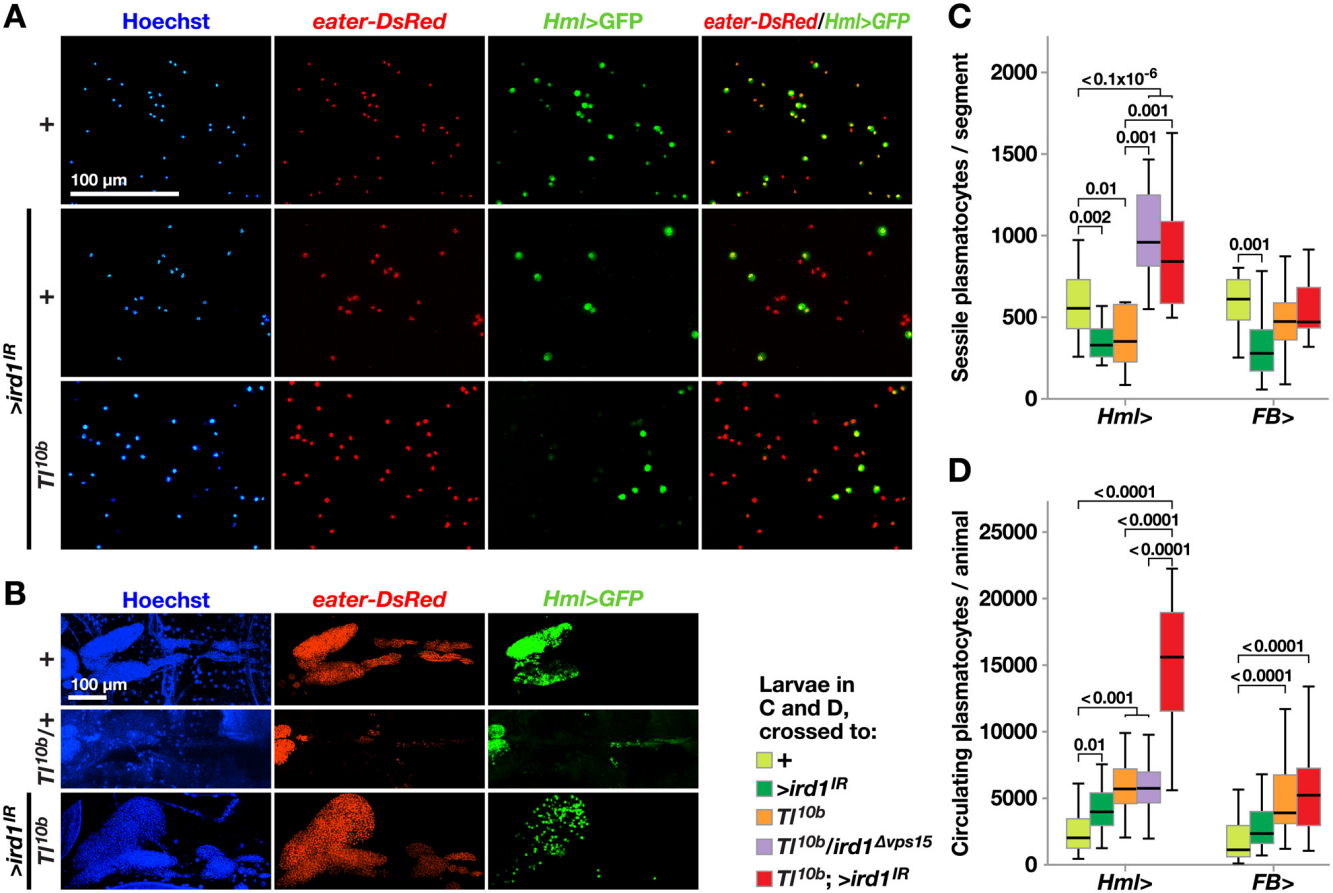
Silencing *ird1* in hemocytes reduces the fraction of *Hml*^*Δ*^>*GFP*–positive cells, while increasing total blood cell numbers and rescuing lymph gland morphology in *Tl*^*10b*^ larvae. **A.** Circulating blood cells are visualized by *Hml*^*Δ*^*-GAL4*-driven *UAS-GFP* (green) and *eater-DsRed* (red) plasmatocyte reporter. Overexpression of *UAS-GFP* alone (+), or together with *UAS*-*ird1*^*IR*^ (>*ird1*^*IR*^) in wild-type (+) or *Tl*^*10b*^ genetic background. **B.** Dissected lymph glands from larvae of the indicated genotypes (the anterior end to the left). Nuclear Hoechst staining (blue) and *eater-DsRed* expression visualize the entire organ, whereas the *Hml*^*Δ*^*>GFP* signal is seen mostly in primary lobes. Between 8–10 lymph glands per genotype were analyzed. **C, D.** Number of *eater-DsRed* expressing sessile plasmatocytes in a single body segment (C) or extracted from circulation of larvae (D), carrying *Hml*^*Δ*^*-* or *FB-GAL4* alone (+), together with either *UAS-ird1*^*IR*^ (>*ird1*^IR^), *Tl*^*10b*^, *ird1*^*Δvps15*^ or both of the latter. Plasmatocytes of the same blood cell populations were also counted for larvae carrying *UAS-Tl*^*10b*^ alone (>*Tl*^*10b*^) or in combination with *Hml*^*Δ*^*-GAL4*. Box-plots in C depict medians and quartiles for number of sessile cells counted automatically using imaging software in microscopic images of 12–15 individual larvae per genotype. For D, 30 larvae per genotype were bled and plasmatocyte numbers assessed using a hemocytometer. Values for significant differences from pairwise comparisons by post-hoc tests after two-way ANOVA are depicted above the whiskers in C and D. Hemocytes in A were imaged by fluorescence microscopy, whereas lymph gland images in B are maximum intensity projections of *z*-stacks generated by confocal microscopy.

The interpretation of these effects is complicated by the fact that suppression of *ird1* in hemocytes also affected the expression of the plasmatocyte-specific marker, *i*.*e*. the proportion of hemocytes that express *Hml*>*GFP*. This effect was clearly observed among circulating hemocytes, where *ird1* suppression reduced the percentage of *Hml*>*GFP*-expressing cells (Fig 3A and quantified in S3B Fig), and in the lymph glands (Fig 3B). We therefore used the *eater-DsRed* marker to re-investigate the effect of suppressing *ird1* in hemocytes. This marker was less strongly affected by *ird1* suppression and visualized most or all plasmatocytes (Fig 3A). In this way we could confirm the rescue of the disrupted bands of the *Tl*^*10b*^ phenotype by the *ird1*^*Δvps15*^ deletion mutant, and show that *Hml*-driven *ird1* RNAi was alone sufficient to suppress this *Tl*^*10b*^ phenotype (S3C Fig). Fig 3B also shows that *Hml*-driven expression of *ird1*^*IR*^ rescued the morphology of the primary lymph gland, which was disrupted in the *Tl*^*10b*^ mutant. To accurately quantify the rescue of the sessile hemocyte pattern we counted the number of *eater-DsRed*-positive hemocytes in the same single segment of each larva (Fig 3C, *cf*. S3C Fig), as well as the number of circulating hemocytes (Fig 3D). In agreement with our previous estimates with the mobilization index assay, *Tl*^*10b*^ larvae had a significantly reduced number of *eater-DsRed*-positive hemocytes in the sessile compartment (compare Figs 2A and 2B and 3C). Not only was this mobilization of blood cells in *Tl*^*10b*^ larvae reversed by a single copy of the *ird1*^*Δvps15*^ mutant, but the number of *eater-DsRed*-positive sessile hemocytes was even significantly increased compared to the wild-type control. This effect could also be mimicked by expression of *ird1*^*IR*^ in hemocytes with the *Hml*^*Δ*^*-GAL4* driver (Fig 3C, S3C Fig), confirming the role of *ird1* for this *Tl*^*10b*^ phenotype. Furthermore, suppression of *ird1* expression in hemocytes significantly increased the number of blood cells in circulation of *Tl*^*10b*^ larvae (Fig 3D), an effect that was not seen in *ird1*^*Δvps15*^ heterozygotes. Thus, the number of hemocytes was increased in both the sessile and the circulating compartments, suggesting a proliferative response.

We also investigated the effect of suppressing *ird1* in hemocytes in the absence of the *Tl*^*10b*^ mutation. This reduced the number of plasmatocytes in the sessile compartment by more than 40%, as detected with the *eater-DsRed* reporter, whereas the number of plasmatocytes in circulation almost doubled (Fig 3C and 3D). In transheterozygous *ird1*^*5*^/*ird1*^*Δvps15*^ (*ird1*^-/-^) and larvae expressing *ird1*^*IR*^ with two hemocyte drivers, *Hml*^*Δ*^*-GAL4* and *He-GAL4*, the number of hemocytes in circulation was also significantly increased (S3D Fig), confirming our results with *Hml*^*Δ*^*-GAL4* alone.

To test if *ird1* activity in the fat body may also contribute to the blood cell phenotypes, we used the fat body-specific *FB-GAL4* driver to express *ird1*^*IR*^, in a *Tl*^*10b*^ or wild-type genetic background. This had no significant effect on the number of sessile cells or plasmatocytes in circulation in *Tl*^*10b*^ animals (Fig 3C and 3D). However in *Toll* wild-type animals the number of sessile cells was reduced and there was also a corresponding increase in the circulating population, although the latter effect was not statistically significant. Combining the *Hml*^*Δ*^*-GAL4* and *FB-GAL4* drivers did not have a stronger effect than the *Hml*^*Δ*^*-GAL4* driver alone (S3D Fig), giving further support to the notion that the *ird1* gene product is not required in the fat body for the *Toll*-dependent blood cell phenotypes.

In conclusion, suppression of *ird1* in hemocytes is sufficient to enhance melanotic nodule formation in *Tl*^*10b*^ larvae to a similar extent as in *ird1* mutants, and it also has a strong but more variable effect on the dispersal of sessile blood cells as well as hemocytes in the lymph gland and on the number of hemocytes in circulation. Thus, the hemocyte phenotypes of *ird1* mutants in *Tl*^*10b*^ larvae can largely be explained by the function of *ird1* in the hemocytes themselves.

### Loss of *ird1* expression in hemocytes triggers lamellocyte formation, and this effect is enhanced by starvation

**Fig 4:**
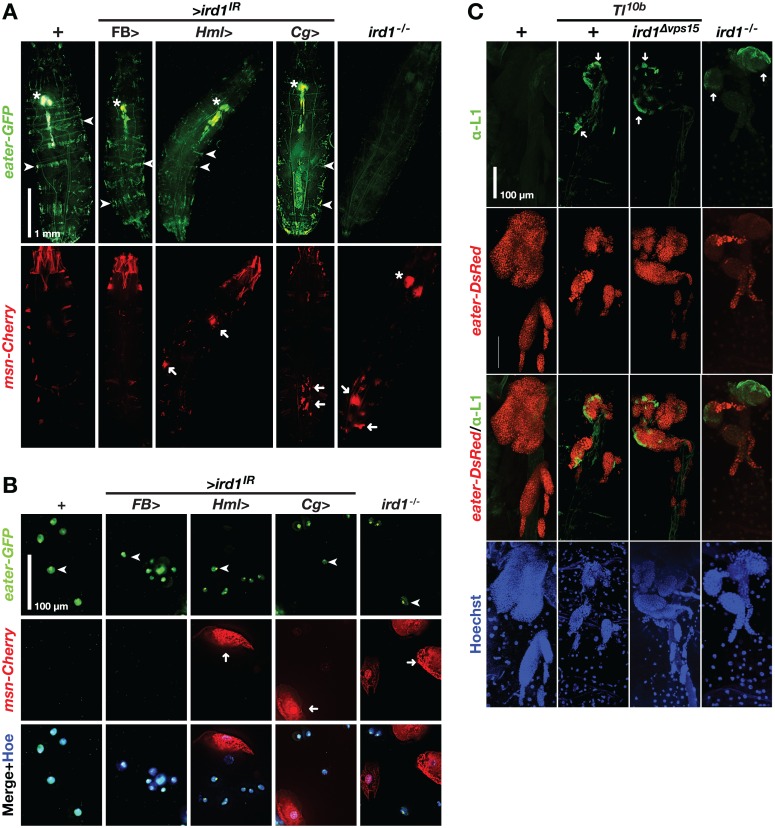
*ird1* loss-of-function in hemocytes triggers lamellocyte formation. **A.** Effects of *ird1* suppression or loss of function on the distribution of hemocytes. Upper panels show plasmatocytes, as visualized with the plasmatocyte marker *eater-GFP*. Lower panels show lamellocytes in the same animals, as visualized with the *msn-mCherry* marker. Strong ectopic expression of the latter marker is also seen in parts of the larval musculature, especially in the head region. Arrowheads: sessile hemocyte bands; asterisks: lymph glands; arrows: nodules. **B.** *eater-GFP* and *msn-mCherry* marker expression in plasmatocytes (arrowheads) and lamellocytes (arrows) extracted from the circulating blood cell population of larvae having the same genotypes as in A. **C**. Effects of *ird1* loss of function on hemocyte distribution in lymph glands of *Tl*^*10b*^ and *Tl* wild-type larvae. Plasmatocyte reporter *eater-DsRed* and nuclear Hoechst staining (blue) visualize the entire organ whereas the L1 antibody marks lamellocytes (arrows). Between 8–10 lymph glands per genotype were analyzed. Hemocytes in B are imaged by regular fluorescence microscopy, whereas lymph gland images in C are maximum intensity projections of *z*-stacks generated by confocal microscopy.

As fewer hemocytes expressed the *Hml*>*GFP* plasmatocyte marker when we suppressed *ird1* expression, we thought that some plasmatocytes may have been triggered to develop into lamellocytes, like in the cellular immune response against parasitoid wasps [27,28]. The increased frequency of melanotic nodules seen in the *Tl*^*10b*^ genetic background would also be consistent with such a response. To test this hypothesis directly we recombined two GAL4-independent hemocyte reporters into the same fly stock, MSNF9mo-mCherry (*msn-Cherry*) for lamellocytes [39] and *eater*-*GFP* for plasmatocytes [40]. In control animals, *msn-Cherry* was only expressed ectopically, mostly in parts of the musculature [31]. When we silenced *ird1* expression in hemocytes with the *Hml*^*Δ*^*-GAL4* or *Cg*-*GAL4* drivers we observed *msn-Cherry*-positive hemocyte aggregates, some of them melanized, in many individuals (arrows in Fig 4A, lower panels; quantified in S4A Fig). The *eater*-*GFP*-labeled bands of sessile plasmatocytes were either interrupted or completely absent (arrowheads in Fig 4A, upper panel). These effects were very similar to the *Toll* gain-of-function phenotypes that could be observed with the same markers [31], except that the lymph glands were still intact after *ird1* suppression (asterisks in Fig 4A, upper panel). In contrast, we saw no effect on hemocyte distribution when we suppressed *ird1* in the fat body with the *FB-GAL4* driver (Fig 4A), and few if any *msn-Cherry*-positive aggregates using this or a second fat body-specific driver, *ppl-GAL4* (quantified in S4A Fig). We further studied the *ird1* phenotype in *ird1*^*5*^/*ird1*^*Δvps15*^ (*ird1*^-/-^) null mutant larvae. As previously reported by others [15], only few larvae of this genotype hatched. They were usually smaller in size, their hemocyte phenotypes were more severe, and they always died before the onset of pupariation. Such larvae never showed obvious *eater*-*GFP* expression in sessile blood cells or lymph glands (Fig 4A upper panel). In addition to *msn-Cherry*-positive nodules, some *ird1*^*-/-*^ animals showed reporter expression in large paired structures located in the same relative position as the lymph glands visualized by *eater*-*GFP* in the other genotypes (see asterisks in Fig 4A upper and lower panels).

Suppression of *ird1* with the *Hml*^*Δ*^*-GAL4* driver reduced the intensity of *eater*-*GFP* expression also in the circulating plasmatocytes (Fig 4B, arrowheads), which were accompanied by large and flat *msn-Cherry*-positive cells with typical lamellocyte morphology, but showing no *eater*-*GFP* signal. This phenotype was also evident in blood samples from *ird1*^*-/-*^ larvae, and when ird1 was suppressed in hemocytes and fat body with the *Cg*-*GAL4* driver, but never when we suppressed *ird1* in the fat body only using the *FB-GAL4* driver. We conclude that specific loss of *ird1* expression in hemocytes triggers lamellocyte differentiation. The fact that all hemocytes analyzed expressed at least one of the blood cell type-specific reporters indicates that they are all viable cells despite some of them having very irregular shapes.

We further confirmed the hemocyte identities in some of the tested genotypes by staining with plasmatocyte- and lamellocyte-specific antibodies [41–43]. All blood cells from wild-type control animals showed strong staining with the plasmatocyte-specific Nimrod C1 antibody P1, but this signal was almost absent in cells obtained from the *ird1*^*-/-*^ mutant (S4B Fig) where they were largely replaced by large lamellocytes, as defined by the lamellocyte-specific Atilla antibody L1 (S4C Fig). A similar phenotype was seen in *Tl*^*10b*^ animals, although the *Tl*^*10b*^ lamellocytes were irregular in shape and more often retained eater-DsRed expression (arrowheads in S4C Fig). Although such double-positive cells were also found in hemolymph from *ird1*^*-/-*^ larvae, most hemocytes were bigger and showed L1 staining only (arrow in S4C Fig).

We took the same approach to analyze the lymph glands of *ird1*^*-/-*^, *Tl*^*10b*^ and *Tl*^*10b*^*/ ird1*^*Δvps15*^ heterozygotes. Unlike earlier studies [17,44], we were able to clearly identify areas of lamellocyte formation in the peripheral parts of the lymph glands from *Tl*^*10b*^ animals, as identified with the L1 antibody (see structures next to arrows in Fig 4C). A similar L1 staining pattern was also seen in the *Tl*^*10b*^*/ ird1*^*Δvps15*^ heterozygotes, although the organs dissected from larvae of the latter genotype showed a more wild-type morphology (as seen by *eater-DsRed* expression and Hoechst staining in Fig 4C). This indicates that the rescue of the aberrant *Tl*^*10b*^ lymph gland morphology by *ird1* RNAi in hemocytes (Fig 3B) does not depend on blocked lamellocyte differentiation. Loss of *ird1* was alone sufficient to induce lamellocyte formation, as shown in the *ird1*^*-/-*^ lymph glands (Fig 4C). The latter were rather small, probably due to the retarded growth of these animals. Like in the circulating hemocytes, L1 antibody staining partially overlapped with *eater-DsRed* expression in *Tl*^*10b*^ and *Tl*^*10b*^*/ ird1*^*Δvps15*^, but not in *ird1*^*-/-*^ lymph glands (see merged images in Fig 4C).

The starvation-induced expression of the antimicrobial peptides Metchnikowin and Diptericin, but not Drosomycin, was previously shown to depend on *ird1* [15]. To test if some of the blood cell-related phenotypes we had observed in *ird1* null larvae were also modified by dietary restrictions, we collected early third instar *ird1* null larvae showing only a single small *msn-Cherry* positive nodule and divided them into two groups. One group was kept on standard food, while the other was starved for 3 h as described [15]. Starved larvae showed bigger and more numerous *msn-Cherry*-positive nodules compared to fed animals (arrowheads in S4D Fig). No *msn-Cherry* positive nodules were observed in wild-type control larvae.

### *ird1* is needed for crystal cell formation and the melanization response against parasitoid wasps

**Fig 5:**
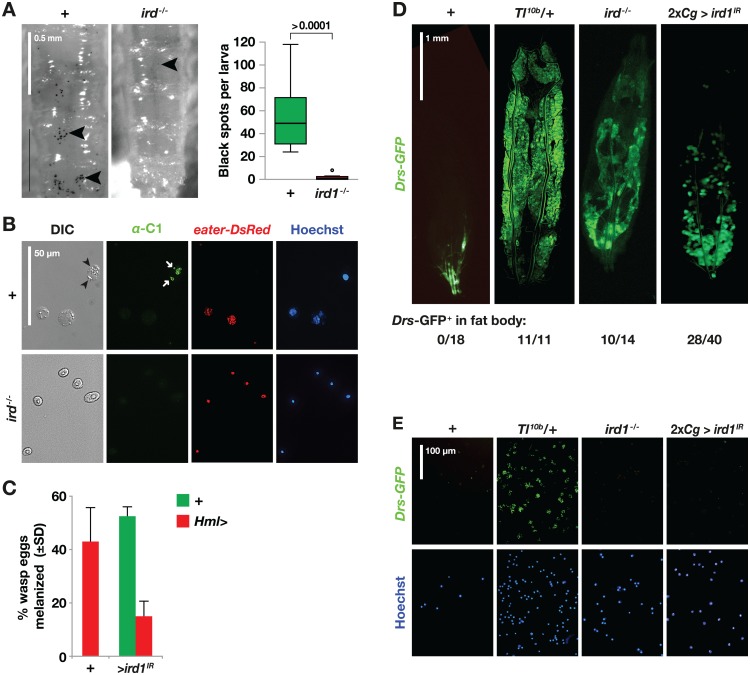
*ird1* loss-of-function prevents normal crystal cell development and activates Toll signaling in the fat body. **A.** Crystal cells in the posterior ends of control (+) and *ird1*^*-/-*^ larvae are visualized (arrowheads) by heat treatment (10 min, 70°C) and quantified. The plots indicate medians, quartiles and outliers for the number of black spots counted in images taken of the dorsal side of eight individuals per genotype. The significance level was estimated by Mann-Whitney U exact test (two-tailed). **B**. Hemocytes from control (+) and *ird1*^*-/-*^ larvae stained with the C1 antibody (green), which is specific for the crystalline inclusions in crystal cells (arrowheads), and Hoechst nuclear dye (blue). The plasmatocyte-specific *eater-DsRed* reporter (red) is never seen in cells that stain with the crystal cell-specific antibody (arrows). **C.** The percentage of larvae with visible black capsules 27–29 hours after infection with the parasitoid wasp *Leptopilina boulardi*. The graph shows the average of two independent experiments with at least 100 larvae for each genotype and trial. **D, E.** Toll signaling activity in larval fat bodies (D) and extracted hemocytes (E), visualized with the *Drs-GFP* reporter in wild-type control (+), *Tl*^*10b*^ heterozygotes (*Tl*^*10b/+*^), *ird1* null mutant larvae (*ird1*^*-/-*^) or animals expressing *UAS-ird1*^*IR*^ (*ird1*^*IR*^) in both fat body and hemocytes with two copies of the *Cg*-GAL4 driver (2x*Cg*>). Values below the images in D indicate the proportion of individuals showing fat body-specific *Drs-GFP* (not counting spontaneous Toll-independent activation of *Drs-GFP* in the trachea).

In a *ird1*^*5*^/*ird1*^*Δvps15*^ (*ird1*^-/-^) null mutant cross, about 60% of the larvae showed melanotic nodules, but when injected with an *E*. *coli* suspension they failed to show melanization at the wound site or on filter papers incubated together with the injured animal (S5A and S5B Fig). These results confirm earlier observations by Wu *et al*. [14], suggesting an impaired function of crystal cells in these animals. Melanin is a product of the phenoloxidase enzymes, and two out of the three phenoloxidase genes in *Drosophila*, *PPO2* and *PPO1*, are specifically expressed in crystal cells. Both genes contribute to injury-mediated melanization, and *PPO2* mutants lack recognizable crystal cells [24]. To test for the presence of crystal cells in the *ird1* mutants, we visualized the crystal cells by heating the animals 10 min at 65°C [45]. As shown in Fig 5A, small black spots of homogeneous size corresponding to single crystal cells can be seen as in all wild-type control animals, mostly restricted to the middle of each larval segment (next to arrowheads and quantified in Fig 5A). In contrast, such spots were absent in *ird1*^*-/-*^ larvae, although a few randomly localized discolorations could be seen, most likely corresponding to small nodules. Using the phenoloxidase-specific C1 antibody [46,47] to look for the presence of crystal cells in the circulating hemocyte population we could detect crystal cells in hemolymph of wild-type larvae, but never in samples collected from *ird1*^*-/-*^ larvae (Fig 5B). Apart from being *eater-DsRed* negative, their crystal cell identity in the wild-type control was confirmed by the co-localization of C1 staining (arrows) with crystalline inclusions (arrowheads, Fig 5B). We conclude that no functional crystal cells, as defined by the presence of phenoloxidase, are formed in the absence of *ird1*.

To determine if *ird1* suppression also affects melanization reactions in a more natural context, we infected second instar larvae with *Hml*^*Δ*^*-GAL4*-driven *ird1*^*IR*^ with the parasitoid wasp *Leptopilina boulardi*. A successful encapsulation response, detected as melanized wasp eggs, was seen in approximately 50% of the control larvae (carrying the driver or the RNAi construct only) 27 h after infection (Fig 5C). In contrast, only 15% of the *ird1*-suppressed larvae were able to mount this type of immune response. Thus *ird1* is required in hemocytes for a full melanization response, in agreement with the role of this gene in the formation and/or function of crystal cells.

### Reduced *ird1* expression activates Toll signaling in fat body but not in hemocytes

Although *ird1* mutations appear to suppress the Toll-mediated mobilization of sessile hemocytes, other *ird1* phenotypes largely overlap with those of the *Tl*^*10b*^ gain-of-function mutant, as described previously by Wu *et al*. [14,15]. The same authors also observed that mRNA of the Toll-responsive *Drosomycin* gene is expressed at an increased basal level in homozygous *ird1* mutant larvae, which suggested to us that activated Toll signaling might be the reason for the Toll-like phenotypes of these mutants. Using the Toll-responsive *Drs*-*GFP* reporter [48], we found that the majority of *ird1*^-/-^ larvae showed a robust GFP expression, similar to the *Tl*^*10b*^ positive controls (Fig 5D), whereas this phenotype was less frequently encountered in heterozygous *ird1*^*Δvps15*^ (*ird1*^+/-^) animals (S5C Fig). This effect seemed to be restricted to the fat body. Specifically we saw no Toll induction in the hemocytes of *ird1*^-/-^ larvae (Fig 5E) although, as previously reported, *Drs-GFP* can be detected in most plasmatocytes and some lamellocytes from larvae that have been septically injured [48]. In the same study it was shown that even wild-type individuals occasionally showed spontaneous *Drs*-*GFP* expression locally in inner organs like the trachea (an example is shown in Fig 5D). We disregarded the latter patterns of tissue expression in our study.

**Fig 6:**
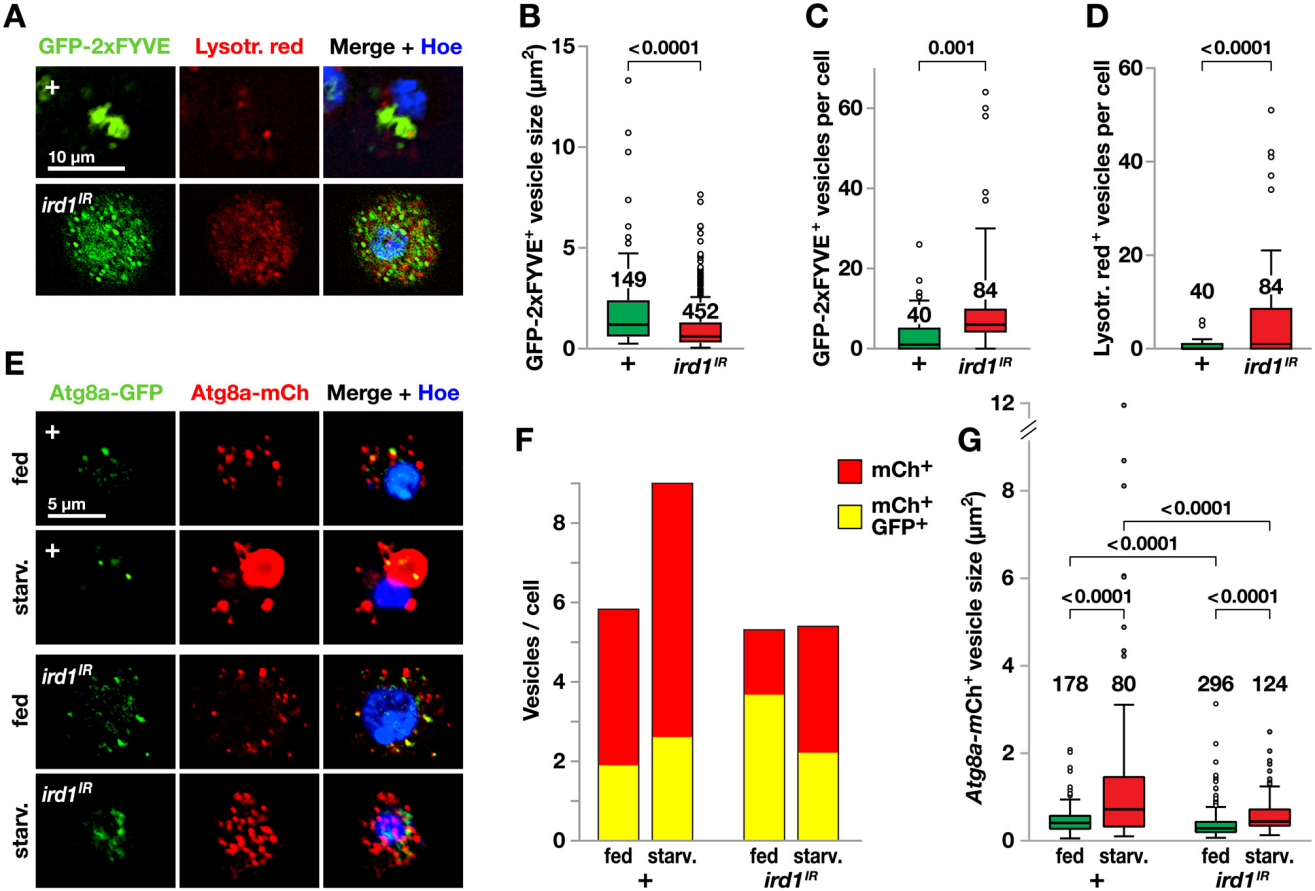
*ird1* expression in plasmatocytes is required for endosome homeostasis and starvation-induced autolysosome formation. **A.** The binding of *Hml*^*Δ*^*-GAL4*-driven *UAS*-*GFP-Myc-2xFYVE* to the product of the Ird1-Vps34 kinase complex visualizes early endosomes and multivesicular bodies (green) in control and *UAS-ird1*^IR^ plasmatocytes from fed larvae only. Hemocytes were also stained with pH sensitive dye Lysotracker red to visualize autolysosomes and with Hoechst. **B-D**. Quantification of sizes (B) and numbers of GFP-staining (C) or Lysotracker red-staining (D) vesicles in images of the indicated number of plasmatocytes from larvae of the same genotypes as in A. **E.** *Hml*^*Δ*^*-GAL4* driven *UAS-mCherry-GFP-Atg8a* visualizes intracellular vesicles in plasmatocytes from control (+) and *UAS-ird1*^*IR*^-expressing larvae that were either starved for 3h or kept on standard food. Hoechst permanently stains the nucleus (blue) while GFP (green) fluorescence is quenched in lysosomes due to the low pH, making the mCherry (red) fluorescence a marker for Atg8a in autolysosomes. GFP (green) fluorescence marks Atg8a presence elsewhere. **F.** The number of vesicles with red only or red and green fluorescence was counted separately for each hemocyte in 7–14 microscopic fields (35–111 cells) per experimental condition. The bars show the average number of vesicles of each category per cell. See S5 Fig for more detail. **G.** Size distribution of mCherry^+^ vesicles, measured in images of the indicated number of plasmatocytes. Box-plots depict medians, quartiles and outliers, and *P* values for pairwise comparisons are indicated above. The latter were calculated using Kruskal-Wallis ANOVA (G) or two-tailed Mann-Whitney U exact tests (B-D).

When we combined *ird1*^*IR*^ with two copies of the *Cg-GAL4* driver, which is expressed both in fat body and hemocytes, we were able to phenocopy the *ird1*^-/-^ mutant-induced activation of Toll signaling in the fat body (Fig 5D), although the signal was only seen in parts of the fat bodies and a single copy of the *Cg-GAL4* driver was not sufficient for this effect (S5C Fig). Like in the *ird1*^-/-^ mutant, the effect was limited to the fat body and no *Drs*-*GFP* expression was detected in hemocytes (Fig 6E). In contrast, expressing *ird1*^*IR*^ with two copies of *Hml*^*Δ*^*-GAL4* or FB-*GAL4* failed to induce *Drs*-*GFP* expression more frequently than driver control animals (S5C and S5D Fig). Thus, it is an open question whether it is the loss of *ird1* function in hemocytes or in fat body that triggers the fat body-specific Toll response, or if *ird1* must perhaps be suppressed in both tissues for this effect. As a positive control, it was sufficient to express >*Tl*^*10b*^ specifically in blood cells with the *Hml*^*Δ*^*-GAL4* driver, in the fat body with the FB-*GAL4* driver, or in both tissues with the *Cg*-*GAL4* driver to activate the *Drs*-*GFP* reporter in the respective tissues (S5C and S5D Fig).

In summary, our results show that the activation of the Toll signaling pathway in *ird1* loss-of-function mutants is restricted to the fat body. The Toll response in the fat body most likely augments the *ird1* hemocyte phenotypes, although Toll signaling in the hemocytes is not involved. However, Toll activation is not strictly required for the *ird1* phenotypes, as some phenotypic effects are seen even when *ird1* is suppressed in the hemocytes (Fig 2C and 2E), when no Toll activation could be detected.

### *ird1* is required for normal vesicle trafficking in the autophagy and endocytosis pathways of plasmatocytes

Using the PI3P binding reporter, *UAS*-*GFP-Myc-2xFYVE*, previous studies have established that class III PI 3-kinase is strictly required for PI3P formation and starvation-induced and developmentally-induced autophagy in the fat body and gut [36,49,50]. In contrast, studies of hemocytes suggest that in this cell type, PI3P can also be produced by the class II PI 3-kinase PI3K68D [51]. Because starvation enhanced the lamellocyte formation phenotype when we suppressed *ird1* in hemocytes, we wanted to find out if *ird1* is required for PI3P formation, autophagy and endocytic trafficking in hemocytes.

To monitor the *in vivo* activity of the PI 3-kinase Vps34 and its controlling subunit Ird1, we used the *UAS*-*GFP-Myc-2xFYVE* construct to visualize PI3P, which is located in early endosomes and multivesicular bodies, and lysotracker, which highlights late Rab7-positive endosomes and lysosomes [52,53]. Hemocyte-specific co-expression of this *GFP-2xFYVE* construct with *ird1*^*IR*^, using the *Hml*^*Δ*^*-GAL4* driver, led to a highly distorted GFP and lysotracker pattern in most plasmatocytes (Fig 6A). In control plasmatocytes PI(3)P was localized to a small number of large structures. Upon silencing *ird1* expression, both GFP-labeled and lysotracker-positive vesicles became smaller, more numerous and highly dispersed within the cytoplasm indicating that the growth and maturation of larger vesicles is blocked in this genotype (Fig 6A–6D). The fact that silencing of *ird1* was not sufficient to remove all PI3P is in line with earlier studies where Vps34 was inactivated in hemocytes, indicating that unlike other cell types, hemocytes can produce PI3P by an alternative pathway, likely by PI3K68D [51].

To monitor autophagy, we took advantage of a *mCherry-GFP-Atg8a* fusion construct that fluoresces green and red in autophagosomal structures, but red only in autolysosomes where the low pH quenches GFP, thus acting as a reporter for autophagy activity and autophagy-mediated flux to the lysosome [54,55]. We expressed this marker with the *Hml*^*Δ*^*-GAL4* driver, in effect limiting our study to plasmatocytes, as we found this driver to be down-regulated in lamellocytes. Plasmatocytes from fed control larvae showed numerous small autolysosomal vesicles marked by mCherry only, whereas a smaller number of vesicles expressed GFP, suggesting that autophagy is active under fed conditions in this cell type (Fig 6E and 6F). Starvation increased the number of vesicles, including the mCherry-only autolysosomes, in control cells (Fig 6F, statistical analysis in S6A Fig), and the mean vesicle size of the autolysosomes increased (Fig 6G). The fraction of mCherry-only autolysosomal vesicles was reduced in *ird1*^*IR*^-expressing plasmatocytes (Fig 6F, statistical analysis in S6B Fig), while the number of double positive structures increased, suggesting that some autophagosomes still formed. In starved animals, *ird1* knockdown effectively reversed the size increase of mCherry-positive autolysosomes, which appeared similar to those of non-starved *ird1*-suppressed cells (Fig 6E and 6G), as well as the total number of vesicles (Fig 6F). Taken together, our results suggest that Ird1 is important for autophagic flux to the lysosome. Surprisingly, double positive mCherry-GFP-Atg8a structures were still present after *ird1* knockdown, supporting the possibility that some autophagosome formation can occur independently of class III PI 3-kinase.

To further confirm the role of vesicular trafficking for the *Tl*^*10b*^ hemocyte phenotypes we tested additional mutants, affecting factors in the same pathways as *ird1* (S7A and S7B Fig). A heterozygous mutation in the *Drosophila* Vps18 homolog, *deep orange* (*dor*^*8*^) [56,57], had the same effects as *ird1*^*Δvps15*^ but mutations in other autophagy genes, including *Vps34* [50], *autophagy-related 1* (*Atg1*) [58], *Atg8a* [59] and *Atg13* [60] only suppressed *Toll*-dependent hemocyte mobilization without affecting nodulation. Besides helping to orchestrate autosome to lysosome fusion, *dor* was suggested to play a conserved role in endocytic trafficking [57,61]. This indicates that while for *Toll*-dependent hemocyte mobilization an undisturbed vesicular trafficking system is of general importance, nodulation may be more specifically regulated by the actions of the endocytosis pathway. Finally, we found that *Tl*^*10b*^ larvae heterozygous for a *phosphatase and tensin homolog* (*Pten*) loss-of-function allele [62] showed both increased nodulation and significantly reduced blood cell mobilization (S7A and S7B Fig), similar to the phenotype of *ird1* mutants. This may seem surprising, since by its function Pten antagonizes the actions of the Ird1-PI 3-kinase complex, but may be in line with our observation that overexpression of ird1 gives a similar phenotype as the *ird1* mutants (Fig 2A and 2C). Thus, an unimpeded vesicle transport is important for normal hemocyte mobilization in response to Toll-activated signals from the fat body.

### Expression of *ird1*^*IR*^ or *Tl*^*10b*^ cause a similar re-localization of the Toll receptor

**Fig 7:**
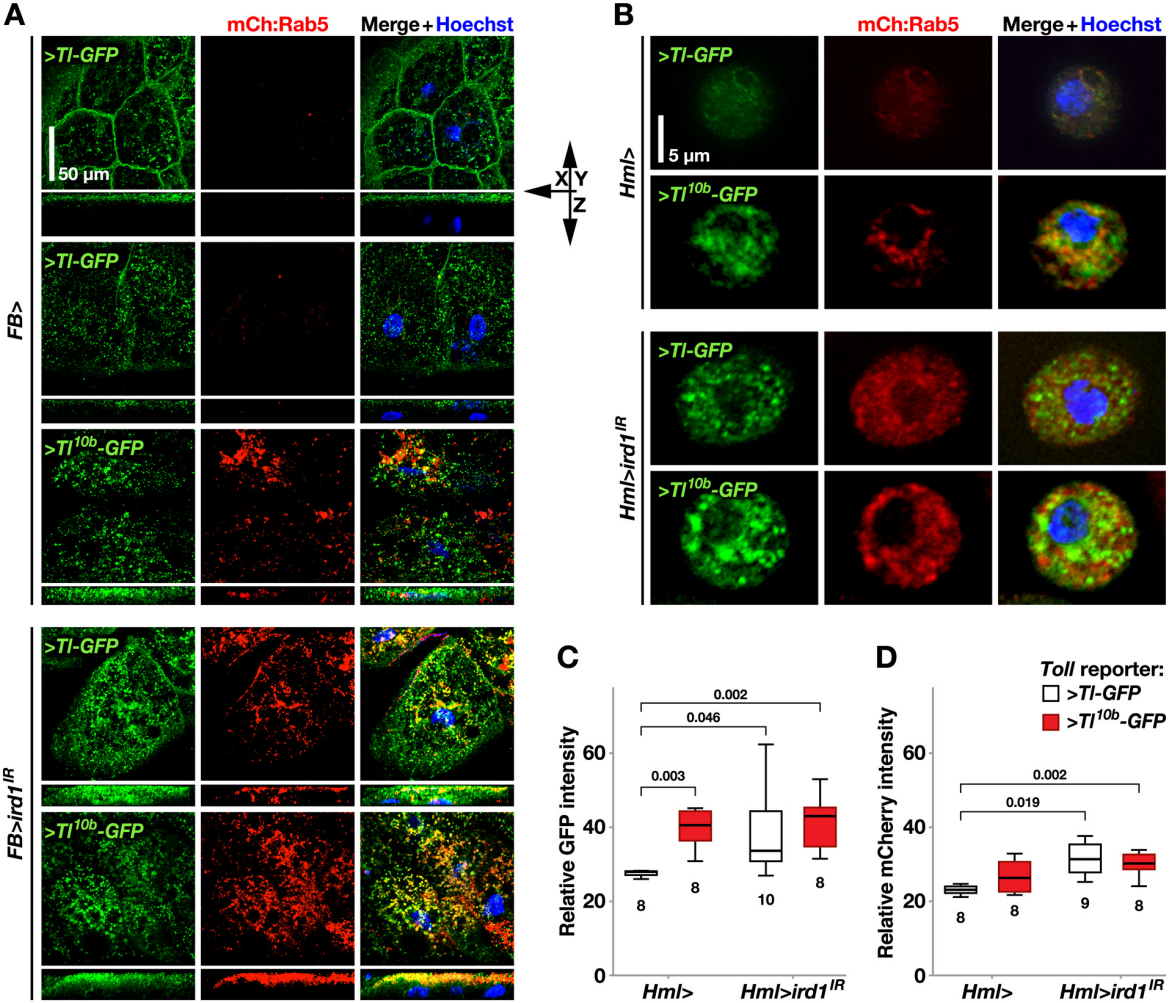
Suppression of *ird1* or activation of Toll signaling cause similar re-localization and accumulation of Toll-GFP fusion protein and early endosomes. **A.** *FB-GAL4* driven *UAS-Tl-GFP* (>*Tl-GFP*) fusion construct and *mCherry*-*Rab5* (*mCh*:*Rab5*) reporter are used to visualize the localization and relative quantity of Toll receptors (green) and early endosomes (red) in fat bodies extracted of otherwise wild-type (+) or *UAS-ird1*^*IR*^-expressing (>*ird1*^IR^) larvae and stained with Hoechst nuclear dye (blue). Using a *UAS-Tl*^*10b*^*-GFP* (>*Tl*^*10b*^*-GFP*) construct, the same structures are also visualized under conditions of an activated Toll pathway. **B.** *Hml*^*Δ*^*-GAL4* driving the expression of the same fusion and reporter constructs, to visualize Toll receptor and early endosome localization in plasmatocytes. **C-D.** Relative signal intensity of GFP (C) and mCherry (D) in hemocyte samples as in B, with either the >*Tl-GFP* (white boxes) or >*Tl*^*10b*^*-GFP* fusion constructs (grey boxes). Box-plots in C and D depict medians, quartiles and outliers measured by software in the indicated number of images, containing 3–12 plasmatocytes each. The *P* values indicated above were calculated for pairwise comparisons using Kruskal-Wallis ANOVA. For fat bodies, maximum intensity projections of *z*-stacks are shown, whereas hemocyte images were made with regular fluorescence microscopy.

In embryos, activated Toll receptors are relocated from the cell membrane to early endosomes [63]. We observed a similar effect when we expressed the *Tl-GFP* fusion constructs of Lund *et al*. [63] in the fat body (Fig 7A, compare row 1 and 3). Like in the embryo, Tl-GFP outlined the cell borders at the plasma membrane in the fat body cells, though in two out of ten preparations the reporter was already partly re-localized from the plasma membrane to vesicles and cytoplasm (Fig 7A, compare row 1 and 2), possibly due to spurious activation of the Toll pathway during dissection. In contrast to *Tl-GFP*, the constitutively active *Tl*^*10b*^*-GFP* construct [63] was seen only in the intracellular compartment (Fig 7A, row 3) and the GFP-positive vesicles were bigger, more numerous and penetrated deeper into cytoplasm. The *Tl*^*10b*^*-GFP* construct also induced the formation of many early endosomal vesicles, as visualized with the *Rab5-mCherry* reporter [63]. However, only a minority of the *Tl*^*10b*^*-GFP* vesicles coincided with the *Rab5-mCherry*-labeled endosomal compartment (see merged images in Fig 7A, row 3).

The number of intracellular *Tl-GFP*-labeled vesicles also increased substantially when we suppressed *ird1* in the fat body and a large number of mCherry-positive vesicles appeared, many of which were also *Tl-GFP*-positive (Fig 7A, row 4). Thus, like in plasmatocytes (Fig 6F) loss of *ird1* expression leads to an abnormal accumulation of endosomal vesicles. The *ird1* phenotype was reminiscent of the effect of the *Tl*^*10b*^*-GFP* construct, although some of the *Tl-GFP* label still remained at the cell membrane in the *ird1*-suppressed fat body cells. Co-expression of *Tl*^*10b*^*-GFP* and *ird1*^*IR*^ with *FB-GAL4* further enhanced the severity of this phenotype, and almost all *Tl*^*10b*^*-GFP* ended up in mCherry-labeled early endosomal vesicles.

Hemocytes are considerably smaller than fat body cells, making it difficult to image the translocation of the Toll receptor. Furthermore, the expression of *Hml*^*Δ*^*-GAL4* can vary considerably from cell to cell. Therefore we averaged the signal intensities over fields with several plasmatocytes in hemolymph samples of the different genotypes. We found that plasmatocytes expressing *Tl*^*10b*^*-GFP* or *ird1*^*IR*^ (together with the *Tl-GFP* reporter) showed stronger GFP expression compared to *Tl-GFP*-only controls (Fig 7B, quantified in C). This difference was not further enhanced by co-expression of *Tl*^*10b*^*-GFP* and *ird1*^*IR*^. In contrast, a constitutively activated Toll pathway alone did not lead to a significant enhancement of the average *mCherry* signal intensity (Fig 7D). Only co-expression of *ird1*^*IR*^ and *Tl-GFP* or *Tl*^*10b*^*-GFP* significantly increased the expression levels of this reporter. Like in the fat body, we saw a similar trend that *ird1* gene silencing in general moderately increased the co-localization of GFP and mCherry in plasmatocytes. In summary, this is one more example that activation of the Toll pathway and suppression of *ird1* expression generate similar phenotypes.

### *ird1* expression in hemocytes is required for protrusion formation

**Fig 8:**
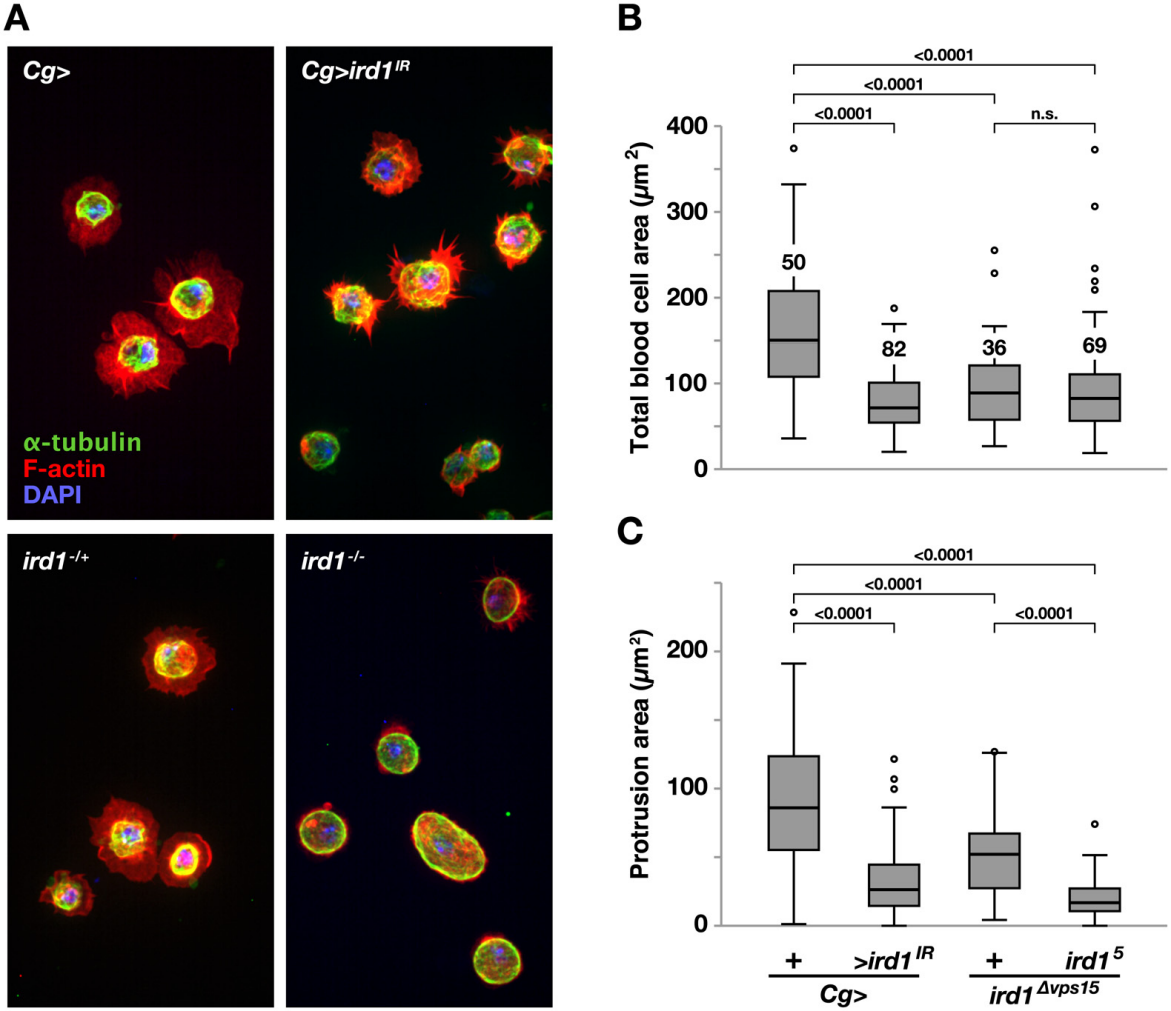
Blood cell protrusion formation requires *ird1*. **A.** Hemocytes expressing *UAS- ird1*^*IR*^ with *Cg-GAL4* (Cg>*ird1*^IR^), driver control hemocytes (Cg>) and hemocytes extracted from *ird1*^*Δvps15*^ heterozygote (*ird1*^*-/+*^) or *ird1*^*Δvps15*^/*ird1*^*5*^ null (*ird1*^*-/-*^) larvae. *Ex vivo* hemocytes spread on glass slides were stained with DAPI nuclear dye (blue), AlexaFluor 594- conjugated F-actin-phalloidin (red) and FITC-conjugated *α*-tubulin antibody (green). **B.** Total area and **C.** protrusion area of hemocytes with the same genotypes as in A. Protrusion area is calculated by subtracting the *α*-tubulin stained region from the total surface spread area stained with phalloidin for each cell using the ImageJ software. Box-plots in B and C depict medians, quartiles and outliers with the number of analyzed blood cells for each genotype indicated in B. *P* values for pairwise comparisons using Kruskal-Wallis ANOVA are indicated above horizontal bars in B and C. Blood cell images are maximum intensity projections of *z*-stacks.

The knockdown of *myotubularin* (*mtm*), another binding partner of Vps34 [64], in *Drosophila* hemocytes was previously shown to give rise to autophagy defects [51] similar to those we observed when we suppressed *ird1* in hemocytes (Fig 6). Further it was demonstrated that increasing or decreasing expression of *mtm* or the autophagy regulator gene *Atg1* in hemocytes hinders blood cell recruitment to wound sites in *Drosophila* larvae [51,65]. The authors argued that the abnormal morphologies displayed by these hemocytes *ex vivo* could explain their immobility. If a reduced *ird1* gene dosage causes morphological defects similar to *mtm/Atg1* knockdown hemocytes, this could explain why many of these cells remain sessile in *Tl*^*10b*^ mutant larvae. Using fluorescently labeled phalloidin to visualize F-actin (red), we found that the total surface spread area of a blood cell was significantly reduced when *ird1*^*IR*^ was expressed with the *Cg-GAL4* driver, compared to control hemocytes (Fig 8A, quantified in B). A similar decrease in average cell area was measured when blood cells were extracted from *ird1*^*-/-*^ or *ird1*^*Δvps15*^ heterozygous larvae. By subtracting the cellular region stained with the α-tubulin antibody (green) from the total phalloidin-labeled cell area (red), we found that the remaining surface F-actin protrusion area was significantly smaller in hemocytes from *ird1*^*-/-*^ null larvae or animals expressing *ird1*^*IR*^ with *Cg-GAL4* (Fig 8C). Unlike a previous study [51], we found that expressing *Vps34*^*KD*^, a kinase-defective version of *Vps34* [50], in hemocytes affected protrusion formation, although the effect was variable. The phenotype of hemocytes expressing *Vps34*^*KD*^ often morphologically resembled that of blood cells with reduced *ird1* gene dosage (two examples shown in S8A Fig). Also, expressing *ird1*^*IR*^ with two hemocyte drivers, *Hml*^*Δ*^*-GAL4* and *He-GAL4*, combined in the same stock with *UAS-GFP*, showed a similar though weaker phenotype as with the *Cg-GAL4* driver (S8B Fig).

No increase in lamellocyte numbers was reported for the *mtm* and *Atg1* mutants, but hemocytes in these mutants were shown to be defective in their ability to reshape and form protrusions [51,65]. We found a similar phenotype in plasmatocytes from the *ird1* mutants, which were unable to spread on a glass surface (Fig 8A, S6B Fig). The periphery of hemocytes obtained from *ird1*^*Δvps15*^ heterozygotes and *ird1*^*-/-*^ larvae, but not *ird1*^*IR*^ expressing larvae, were on average more rounded than those of driver controls, and the effect could be quantified by estimating the circularity of the cells in the images (S6C Fig).

## Conclusions

The artificially elevated activity of the Toll pathway in the *Tl*^*10b*^ mutant has provided a sensitized genetic background, appropriate for a modifier screen, and we found that the hemocyte activation phenotype we scored was indeed affected by heterozygous deletions in numerous chromosomal regions. The sensitivity of the phenotype also led to substantial and significant effects when we crossed our *Hml>GFP*; *Tl*^*10b*^ stock into different wild-type genetic backgrounds. We therefore focused on regions that gave the strongest and most consistent suppressive effects and the results we present here were also confirmed by testing several independent deficiencies, mutants and RNAi experiments.

Although we first identified the *ird1* mutants because of their suppressive effect on the *Tl*^*10b*^ hemocyte mobilization phenotype, they turned out to enhance all other hemocyte phenotypes of the *Tl*^*10b*^ mutant, except the dispersal of primary lymph gland lobes. In fact, the *ird1* null mutant generates *Tl*^*10b*^-like phenotypes even in a *Toll* wild-type background, including lamellocyte formation, melanotic nodules and increased numbers of circulating hemocytes. However, the *ird1* mutants are unique in the way they affect hemocyte behavior. *ird1* mutant plasmatocytes do not spread in a normal way on an artificial substrate, and lamellocytes are not efficiently released from their attachment sites under the larval epidermis. Thus, the apparent suppression of the *Tl*^*10b*^ hemocyte mobilization phenotype may in fact due to a generally reduced mobility in the *ird1* mutant hemocytes. This would also explain why the primary lymph gland lobes do not disperse, although they otherwise show all signs of being activated.

Whereas Toll-dependent activation of hemocytes is cell non-autonomous and mainly dependent on Toll signaling in the fat body [31], the *ird1* mutant phenotypes depend to a large extent on the loss of *ird1* function in the hemocytes themselves. We found that hemocyte-specific knockdown of *ird1* was sufficient to suppress *Tl*^*10b*^-dependent mobilization of hemocytes in subepidermal bands and lymph glands, and to enhance other *Tl*^*10b*^ hemocyte phenotypes. However, there is also contribution from the fat body to the *ird1* null phenotype. It seems likely that Toll signaling becomes activated in the fat body in *ird1* mutants, independently of *ird1* function in the hemocytes.

The molecular mechanisms behind the effects of *ird1* mutants on hemocytes remain unclear. In line with our findings, another component of the PI 3-kinase, Atg6, was recently also shown to generate hemocyte activation phenotypes, including melanotic nodule formation [66]. PI 3-kinase mutants, like *ird1* and *Atg6* are likely to affect several cellular processes, including autophagy and the endocytic recycling of receptors. The same is true for the other vesicle transport mutants we tested, and our experiments do not give a definite answer which cellular process is involved in the phenotypes we have studied. The *ird1*-dependent re-localization of the Toll receptor in the fat body may explain why Toll signaling is activated in that tissue, but it is uncertain to what extent Toll signaling contributes to the *ird1* phenotype. Shravage *et al*. [66] saw no suppression of the *Atg6* melanotic nodule phenotype in heterozygous *Dif*, *dorsal* deficiency larvae that were also *Dif*^*1*^ mutant, but it was not tested if Toll signaling was completely suppressed in these animals.

As the *ird1* gene encodes a PI 3-kinase subunit with a central role in vesicular trafficking, presumably in many cell types, *ird1* mutations might be expected to have very pleiotropic effects. It is therefore striking that phenotypic effects of *ird1* mutants were so far only found in two genetic screens that were both related to immunity: our screen for genes that affect blood cell activation, and the earlier screen by Wu *et al*. [14] for the induction of antimicrobial peptides. We conclude that immune responses place particularly high demands on vesicle transport systems and cellular mobility.

## Materials and Methods

### Fly stocks

Deficiency stocks and *w*^*1118*^iso control flies, all with the same genetic background [33], were originally obtained from the late Szeged *Drosophila* stock center (Szeged, Hungary) and the Bloomington Stock Center (Bloomington, IN). The constitutively active *Toll*^*10b*^ mutant (*mwh*^*1*^ *e*^*1*^ *Tl*^*10b*^ */T(1;3)OR60/TM3*, *Sb*^*1*^ *Ser*^*1*^) [32] and the hemocyte-specific driver *Hml*^*Δ*^*-GAL4* (*w*^*1118*^; *P{Hml-GAL4*.*Δ}2 P{wUAS-2xEGFP}AH2*) [67] were for our purposes combined into one fly line. The *hdc* mutant alleles (*hdc*^*Fus-6*^ and *hdc*^*Fus-6-50*^) and the UAS-*hdc* strain [68,69] were provided by Christos Samakovlis. *pum*^*13*^ is a point mutation encoding a protein that is unable to repress *hb* mRNA translation [70]. *pum*^*3*^ has been used previously [71] but no molecular information has been reported. The loss-of-function allele of *Ptenc*^*494*^ [62] was a gift from Ernst Hafen. Thomas Neufeld provided us with the loss-of-function alleles of *Vps34*^*Δm22*^ [50], *Atg1*^*Δ3D*^ [58] and *Atg13*^*Δ81*^ [60].

Fly stocks carrying *hdc*-RNAi constructs were from the Vienna Drosophila Resource Center (VDRC), with construct ID numbers 39876, 39877, 45069 from the GD and 104322 from the KK collection. *Rab23* alleles, *Rab23*^*51*^ [72] and *Rab23*^*T69*^ [73], were obtained from József Mihály. The *D1* alleles *Df(3R)D1*^*c12w-*^, *Df(3R)D1*^*4A*^, *D1*^*EP473rec#70*^, *D1*^*Rev1B*^ and *Df(3R)D1*^*70-1*^ [74] were provided by Karen Weiler. The five EMS-induced point mutants *ird1*^*1*^—*ird1*^*5*^ and the *P[w*^*+*^, *UAS-ird1 PA10-68]* (here called *ird1*^FL^) insertion line [15] were kindly provided by Louisa Wu. The small deletion *Δvps15* (here called *ird1*^*Δvps15*^) is described elsewhere [36]. A transgenic fly line harboring an inverted *ird1* cDNA fragment under UAS control (here called *ird1*^*IR*^) [38] was provided by Satoshi Goto. A kinase-defective *UAS-Vps34*^*KD*^ construct [50] was obtained from Thomas Neufeld. The *UAS-Tl*^*10b*^ stock (*y w P{UAS-Tl*^*10b*^*}11*), constructed by Jean-Marc Reichhart (Centre National de stock Recherche Scientifique, Strasbourg, France) was a gift from him and it is now deposited at the Bloomington Stock Center. Overexpression of this construct is known to activate the Toll pathway [30,31]. A hypomorphic point mutation of *deep orange*, *dor*^*8*^*/FM6* [18], and the *Atg8a* loss-of-function line *y1 P{SUPor-P}Atg8a*^*KG07569*^*/FM7c* [66] were obtained from the Bloomington Stock Center.

The following tissue-specific drivers were used to create specific loss-of or gain-of-function phenotypes in larvae, using the GAL4-UAS technique [75]: *Hml*^*Δ*^*-GAL4*, alone or in combination with another blood cell-specific *GAL4* line *He-GAL4* (*P{He-GAL4*.*Z}*) [30], the fat body-specific drivers *FB-GAL4* (*P{GAL4}fat*) [76,77] and *ppl-GAL4* [78,79] and the hemocyte- and fat body-specific driver *Cg-GAL4* (*w*^*1118*^*; P{Cg-GAL4*.*A}2*) [80]. FB-GAL4 was obtained from the former Umeå Drosophila Stock Center. All other GAL4 lines can all be obtained from the Bloomington Drosophila Stock Center, and except for the *ppl* driver, we have checked their tissue specificities [31].

The following hemocyte type-specific reporter lines were used in the present study: for plasmatocytes *eater-GFP* [81] and *eater-DsRed* [39], and for lamellocytes *MSNF9mo-mCherry* (hereafter called *msn-Cherry*) [39]. They were obtained from the lab of Robert Schulz. For fat body *in vivo* and blood cell *ex vivo* observation of Toll activation, the *Drs-GFP* reporter (P{Drs-GFP.JM804}1) [48] was obtained from Dominique Ferrandon. To monitor the localization of the Tl-receptor in blood cells and fat bodies with and without a constitutively active Toll pathway, we expressed *UAS-Tl-GFP* and *UAS-Tl*^*10b*^*-GFP* constructs [63] provided by Robert DeLotto.

To visualize intracellular vesicles in hemocytes, we used the following reporter lines: *UAS-mCherry-GFP-Atg8a* [54] to monitor autophagosomes and lysosome fusion, the *UAS*-*GFP-Myc-2xFYVE* construct [53] for analysis of binding to membranes of early endosomes and multivesicular bodies and *mCherry-Rab5* [63] to mark early endosome in both blood and fat body cells.

### Fly crossing and handling of larvae

For each experimental cross 20 virgin females and 5–10 males of the desired genotypes were confined into a bottle containing standard potato diet with yeast and, when needed for staging, red household food dye. Crosses were transferred into new bottles every other day and kept in an incubator at 26°C and 60% humidity. When genes were to be silenced or over-expressed in the larval offspring, culture bottles were kept at 29°C to ensure optimal efficiency of the UAS/GAL4 system. Larvae were collected from bottle walls after 3–6 days and staged according to published procedures [82] at a stage just before the gut contents were completely cleared. To prepare for *in vivo* inspection or dissection, the collected larvae were gently washed and either transferred to water-containing Petri dishes and kept on ice, or directly ripped open on multiwall slides for hemocyte collection.

### Nodule frequency and grading of sessile hemocyte banding pattern

Before analysis, bottles containing the crosses were assigned with arbitrary numbers to blind the examiner, and the genotypes of the cross were not revealed before completion of the experiment. For assessing nodule frequencies, for each cross 50 F1 third instar larvae were collected at random, gently washed in water with a paintbrush and then inspected for nodule frequencies under a stereo microscope. To grade the banded pattern of sessile hemocytes, additional larvae were collected and laid with their ventral side down on ice-cold glass slides. The larvae were then embedded in 50% ice-cold glycerol under a cover glass before being transferred into a refrigerator. After 20 min at -20°C or over-night incubation at +4°C, they were placed on ice and immediately analyzed under a fluorescence microscope. For each deficiency tested in the screen, 16–22 larvae from 1–5 independent crosses were individually scored for the degree of mobilization of the bands of sessile hemocytes under the epidermis [35]. A mobilization index was defined as follows: Grade 1, larvae with sessile hemocyte bands in all segments; Grade 2, and 3, bands of sessile cells in less than 50 and 75% of the segments respectively; Grade 4, no discrete bands of sessile cells in any, or at most in the posterior 25% of the segments (see Fig 1A). In other experiments, all crosses were repeated three times and the nodule frequencies and the average mobilization indexes were calculated each time.

### Infection by bacteria and parasitoid wasps

Third instar larvae were infected with an *Escherichia coli* (*E*. *coli*) suspension and the following melanization reactions monitored as described [14]. Fly crosses for wasp infection were performed as described above, except that they were transferred daily into new vials kept at 25°C. At day three after egg-lay, 20 female and 10 male *Leptopilina boulardi G486* wasps were transferred to the culture. After 2 h at room temperature the wasps were removed and the vials transferred to an incubator set to 29°C and 60% humidity. After 25 hours in the incubator, third instar larvae were collected and dissected under a stereo microscope until approximately 100 infected larvae were scored. If this number was not achieved within two hours, the procedure was repeated the next day with parasitized offspring produced by the same cross. A wasp egg was counted as melanized if it showed any discoloration.

### Blood cell counting

To collect blood cells, 10 third instar larvae per cross were placed separately in the wells of a 12-well glass slide, each containing 20 μl of ice-cold phosphate-buffered saline (PBS; 137 mM NaCl, 2.7 mM KCl, 6.7 mM Na_2_HPO_4_, 1.5 mM KH_2_PO_4_). The animals were carefully ripped open with the help of two watchmaker forceps and the carcasses were removed from the glass slide and 10 μl of the blood cell suspension were transferred to a Neubauer hemocytometer chamber (Paul Marienfeld GmbH & Co. KG, Lauda-Königshofen, Germany). Plasmatocytes were recognized based on their morphology and (if present) by the expression of blood cell type-specific reporters. Counting was done using a phase contrast microscope, equipped with a UV source (Axioplan, Carl Zeiss, 40–60 x magnification) and each experiment was repeated three times using larvae produced by independent crosses.

### Specific staining of hemocyte classes

The specificity of expression of GAL4 lines and reporters for different hemocyte types was tested using mouse monoclonal primary antibodies. To identify plasmatocytes we used P1/ Nimrod C1 and for lamellocytes L1/Atilla antibodies [42] both provided by István Andó. Crystal cells were detected using the C1 (HC12F6) antibody [46,47], a kind gift of Tina Trenczek. Crystal cells were also visualized by heating larvae in water filled tubes for 10 min in a heat block set to 65°C [45]. Primary antibodies were visualized with Cy2-conjugated goat anti-mouse polyclonal antibody (1:600, Millipore), Cy3 conjugated AffiniPure Goat Anti-Mouse IgG (H+L) (1:2000, Jackson ImmunoResearch) or goat anti-rabbit IgG conjugated to Cy3 (1:3000, Life Technologies), all diluted in PBS with 3% bovine serum albumin (BSA). To visualize blood cell morphology, cells were stained with anti-α-tubulin-FITC antibody (1:100, Sigma-Aldrich) and/or AlexaFluor 594 Phalloidin stain (1:50, Life Technologies) and 4',6-diamidino-2-phenylindole (DAPI, 1 μg/ml, Sigma-Aldrich) diluted in PBS.

### Blood cell, fat body and lymph gland preparation and staining

For hemocyte preparation, larvae were opened as described above, carcasses removed and the glass slides containing the hemocytes transferred to a humid chamber to let the blood cells attach for 20–30 min at room temperature (RT). To expose fat body or lymph glands, larvae were sliced open laterally in PBS using fine scissors and as much of the unwanted internal organs as possible were removed. The carcasses were then transferred separately into wells of a 12-well glass slide containing PBS on ice. From this step onward blood cell- and carcass-containing wells were treated the same way. Next, PBS was removed and replaced by 3.5% paraformaldehyde (Sigma-Aldrich) in PBS. After 10 min of fixation, wells were washed three times for 5 min with PBS. After the last PBS removal, 0.1% Triton X-100 (Sigma-Aldrich) in PBS was added to permeabilize cell membranes. After 10 min, the PBS washing step was repeated and 3% BSA in PBS added. After 1 h at 4°C the BSA solution was exchanged for the respective primary antibody solution, diluted with 3% BSA. Following an overnight incubation at 4°C and a PBS washing step, secondary antibody diluted 1:2000 in 3% BSA was added and the cells were incubated in the dark for 1 h at room temperature, then washed twice with PBS, and finally a Hoechst 33258 (Sigma-Aldrich) or DAPI solution (both 1 μg/ml, Sigma-Aldrich) in PBS was added for 10 min. After a final washing step the wells were mounted with 50% glycerol in PBS or Prolong Gold Antifade Reagent (Life Technologies) before sealing the sides of the cover glass with nail polish for microscopy. For visualization of lysosomes, hemocytes were bled into Schneider’s Drosophila medium (Gibco) and stained for 5–7 min at room temperature in the dark with a solution containing Hoechst and LysoTracker red DND-99 (0.03 μg/ μl, Invitrogen) in anhydrous dimethyl sulfoxide (1 mg/100 ml; Sigma-Aldrich). The cells were then washed, fixed and mounted as described above.

### Quantitative RT-PCR

Total RNA was prepared of hemocytes collected as described above using the Aurum Total RNA Mini Kit (BioRad). One-step quantitative real-time PCR was performed in duplicate using the probe detection system as described [83] with an I-cycle iQ Thermal Cycler (BioRad). The following primers were used: sense, 5’-TTCTGCATGAGCAGGACCTC-3’; antisense, 5’-GGTTACGGATCGAACAAGCG-3’ for Rpl32 (Ribosomal protein large subunit 32) that functioned as a control and sense, 5’-GACTATCTCAACATGAACTAA-3’; antisense, 5’-TTAGTTCATGTTGAGATAGTC-3’ for the *hdc* gene. Reactions were set up in a total of 25 μl per sample: 12.5 μl BioRad (iScript One-Step RT-PCR Kit with SYBR Green. The exact contents of this kit appear to be a company secret), 10 μl H2O, 0.5 μl of each *hdc* sense and antisense primer (10 μM) and 0.5 μl reverse transcriptase enzyme (BioRad, the enzyme activity appears to be a company secret). The same volumes were used for Rpl32. Cycle conditions were as follows; cDNA synthesis (50°C for 10 min), denaturation (95°C for 3 min) and PCR cycles (95°C for 10 s, 60°C for 20 s and 72°C for 20 s) repeated 40 times.

### Microscopy and image analysis

Grading of larvae was done with an Axioplan light and UV microscope (Carl Zeiss, 2.5–10 x magnification). Images of living larvae or blood cells stained with antibodies and expressing reporters were taken with the same set up, or with a Nikon Digital sight color camera (Ds-Fi1), on a Nikon Eclipse 90i microscope run by NIS-Elements AR software. The same computer program was used to improve image quality by 2D real-time deconvolution and to quantify intracellular vesicle sizes and relative signal intensities or co-localizations. Sessile blood cell numbers were quantified automatically in cropped and inverted 8-bit microscopic images of the same segment in all larvae (10 x magnification), which had proven to be most reliable, with the ITCN plugin module of the ImageJ software (version 1.47; http://imagej.nih.gov/ij/). Lymph glands and fat bodies were scanned at an optimized number of slices using a Nikon laser confocal microscope (D-Eclipse C1) and images converted into maximum intensity projections using the NIS-Elements AR software. In addition, all images were enhanced using Photoshop CS3 software. For *ex vivo* imaging of hemocyte morphology, stained cells were imaged using a Nikon Eclipse T*i* confocal microscope and Andor iQ3 software. Z stack images with 100x magnification were taken separately for three channels (green, red, blue). Z projection images were generated and merged images including all three channels were created using ImageJ. *Ex vivo* hemocyte spread areas on glass slides were also quantified using ImageJ.

### Statistical analysis

A Kruskal-Wallis one-way analysis of variance (ANOVA) by ranks test was run to automatically compare distributions across groups determining significant differences in pairwise comparisons of mobilization indices as well as, sizes, numbers and relative proportions of intracellular vesicles. Mobilization indices were calculated for repeated crosses of the same genotype as well as different genotypes. When only two groups were compared the Mann-Whitney U exact test was applied. Data from sessile and circulating plasmatocyte counts were log10 transformed and then analyzed for significant differences using ANOVA with a Games-Howell post-hoc test (equal variances not assumed). All statistical data analysis was done with the IBM SPSS software, version 22.

## Supporting Information

S1 Fig Deletions over the strong suppressor regions 1 and 2, and mutant alleles of *Rab23*, suppress *Tl*^*10b*^-induced loss of hemocyte bands. **A.** Mapping of suppressive region 1, using Exelixis (Exel) and DrosDel (ED) deletions, to the region highlighted in light grey. Horizontal lines indicate the lengths (in kb) and relative positions of strong suppressive (red) and weak or non-suppressive deficiencies (black). Green and red dashed lines indicate the mobilization indices calculated for wild-type (+) and *Tl*^*10b*^ mutant control crosses, respectively. **B.** Average mobilization indices, calculated from all scored heterozygous *Rab23* mutant larvae and the corresponding controls. The total numbers of analyzed individuals and the standard deviations are indicated. Values above the bars represent significance levels from pairwise comparisons to the average mobilization index of *Tl*^*10b*^ mutant control crosses, as estimated using Kruskal-Wallis ANOVA. **C.** Mapping of suppressor region 2, presented as in panel A. **D.** Average mobilization indices, calculated from all scored larvae of selected mutants, deficiencies and controls from suppressive region 2, presented as in panel B.
(PDF)

S2 Fig Loss-of-function alleles and hemocyte-specific RNAi attribute the suppressor effect of *Df6332* to *hdc*. **A.** Sessile hemocytes, visualized with *Hml*^*Δ*^*-Gal4*-driven *UAS-GFP*, in wild-type control (+) or mutant larvae, carrying either *Tl*^*10b*^ alone or combined with *Df6332* or *hdc* loss-of-function alleles (*Fus6* or *Fus6-50*). The same driver is used to express different UAS-*hdc* RNAi (*hdc*^IR^) constructs (39876GD, 39877GD, 45069GD and 104322KK) in hemocytes of *Tl*^*10b*^ larvae, or a full-length *hdc* construct (*hdc*^FL^) in *hdc*^*Fus6-50*^*/ Tl*^*10b*^ heterozygote genetic background. The larvae are oriented with the anterior end up. The bar below each image represents the average mobilization index calculated from the indicated number of larvae of the same genotype. *P* values were calculated from pairwise comparisons with *Tl*^*10b*^ mutant control cross, using Kruskal-Wallis ANOVA test. **B.** Suppression of *hdc* expression in hemocytes by different UAS-*hdc*-RNAi constructs, compared to the *Hml*^*Δ*^*-Gal4* driver alone, assayed in hemolymph by quantitative PCR.
(PDF)

S3 Fig Hemocyte phenotypes of *ird1* mutants and in *ird1*-suppressed larvae, with or without the *Tl*^*10b*^ allele. **A.** Mean fraction of larvae with at least one melanotic nodule in three independent crosses, with 50 inspected larvae per indicated genotype and cross. **B.** Mean fraction of circulating blood cells that express *Hml*^*Δ*^*-Gal4*-driven *UAS-GFP* fluorescence in hemocyte smears from larvae that carry the driver, alone or together with *UAS*-*ird1*^*IR*^ (>*ird1*^*IR*^) in wild-type (+) or *Tl*^*10b*^ genetic background. The number of analyzed images is indicated within the bars and the significance level as estimated by Mann-Whitney U exact test (two-tailed) above. **C.** The plasmatocyte-specific reporter *eater-DsRed* visualizes the pattern of sessile cells in third instar larvae heterozygous for the *Tl*^*10b*^ allele (*Tl*^*10b/+*^), alone or in combination with either the heterozygous *ird1*^*Δvps15*^ allele (*ird1*^*Δ/+*^) or *Hml*^*Δ*^*-Gal4* (*Hml>*) driven *ird1*^IR^, compared to the wild-type control (+). The *UAS-GFP* construct was present on the same chromosome as the *Hml*^*Δ*^*-Gal4* driver, but the green channel is not shown here. The white frame indicates the segmental area used to quantify the number of sessile cells in Fig 3. **D.** The number of hemocytes in hemolymph from *ird1* null (*ird1*^*5*^/*ird1*^*Δvps15*^) larvae compared to heterozygous controls (+/*ird1*^*Δvps15*^) and larvae carrying *Cg-GAL4* (Cg>) or *Hml*^*Δ*^*-Gal4* (*Hml>*) combined with either *He-GAL4* (*Hml>He>)* or *FB-GAL4* (*Hml>FB>*) drivers, alone or in combination with *ird1*^*IR*^. Box-plots show medians, quartiles and outliers of hemocyte numbers from 10 larvae per genotype. Significance values for differences as estimated by pairwise comparisons using Kruskal-Wallis ANOVA test. Non-significant differences are not indicated.
(PDF)

S4 Fig *ird1* hemocyte phenotypes as seen with *msn-Cherry*, *eater-DsRed*, and antibody markers. **A.** Fraction of *ird1* null (*ird1*^*-/-*^) or *ird1* RNAi (>*ird1*^IR^) larvae showing at least one blood cell accumulation visualized by the *msn-Cherry* lamellocyte reporter. The *UAS-ird1*^*IR*^ (>*ird1*^IR^) construct was expressed with hemocyte (*Hml>*), fat body (*FB>* and *ppl>*), fat body+hemocyte (*Cg>*), or ubiquitous (*da>*) drivers. The number of analyzed individuals of each genotype is indicated. **B-C.** Hemocytes from wild-type (+), *Tl*^*10b*^ heterozygous (*Tl*^*10b/+*^), or *ird1* functional null (*ird1*^*-/-*^) larvae, carrying the *eater-DsRed* plasmatocyte reporter, stained with Hoechst nuclear dye (blue) and (B) plasmatocyte- or (C) lamellocyte-specific antibodies (green). Arrowheads and arrows in C indicate lamellocytes that have retained or lost *eater-DsRed* expression, respectively. **D.** Expression of the *msn-mCherry* lamellocyte marker in control (+) and *ird1*^*-/-*^ larvae, before and after starvation. Arrowheads indicate lamellocyte accumulations. The *msn-mCherry* marker was also strongly expressed ectopically in parts of the larval musculature.
(PDF)

S5 Fig *ird1* null mutant larvae have melanization defects. *ird1* heterozygotes but not *ird1*^*IR*^ animals show Toll pathway activation in the fat body. **A.** Spontaneous melanotic nodule formation (arrowhead) in an *ird1*^*5*^/*ird1*^*Δvps15*^ (*ird1*^*-/-*^) transheterozygous larva. **B.** Posterior parts of wild-type control (+) and *ird1*^*-/-*^ larvae, 4 h after injection with an *E*. *coli* suspension (upper panels), and filter paper incubated together with the infected animals (lower panel). **C and D.** Toll pathway activation, as detected by the *Drs-GFP* reporter, in larval fat bodies (C) or extracted hemocytes (D) of *ird1*^*Δvps15*^ heterozygotes (*ird1*^*+/-*^), or in larvae expressing *UAS-Tl*^*10b*^ (>*Tl*^*10b*^) or *UAS-ird1*-RNAi (>*ird1*^IR^) in fat body alone by the *FB-Gal4* (*FB>*) driver, or in fat body and hemocytes by the *Cg-GAL4* (*Cg>*) driver. *Drs-GFP* expression is also visible in sessile (C) and circulating (D) blood cell populations of larvae expressing *UAS-Tl*^*10b*^ in hemocytes only by the *Hml*^*Δ*^*-Gal4* (*Hml>*) driver.
(PDF)

S6 Fig *ird1* knockdown blocks the starvation-induced increase in autophagosome and autolysosome numbers in hemocytes and reduces the fraction of hemocyte autolysosomes in fed larvae. **A.** Total number of *Atg8a*-GFP and/or *Atg8a*-mCherry-labeled vesicles per cell. **B.** The fraction of vesicles in each cell that was labeled with *Atg8a*-mCherry only. Each dot represents the count from a single cell and the black bars indicate median values. *P* values for pairwise comparisons using Kruskal-Wallis ANOVA test are shown above.
(PDF)

S7 Fig Modification of *Tl*^*10b*^ phenotype by mutants of vesicle transport genes. **A**. Average mobilization index and **B** percentage of animals expressing *UAS-GFP* with blood cell specific *Hml*^*Δ*^*-Gal4* (*Hml>*) driver with at least one melanotic nodule among wild-type control, or *Tl*^*10b*^ larvae with or without the indicated loss-of-function alleles. Three independent experiments were performed for each genotype, 20 larvae were graded and 50 were inspected for nodules in each. Significant difference (***, *p*<0.0001; **, *p*<0.001) compared to the *Tl*^*10b*^ mutant control, as estimated by pairwise comparisons using Kruskal-Wallis ANOVA test. Non-significant differences are not indicated.
(PDF)

S8 Fig Effects of *Vps34* and *ird1* suppression on hemocyte morphology. **A.** Two examples of hemocytes expressing *UAS-Vps34*^*KD*^ with *Cg-GAL4* (*Cg*>*Vps34*^*KD*^) spread on glass slides and stained with DAPI nuclear dye (blue), AlexaFluor-conjugated phalloidin (red) and FITC-conjugated α-tubulin antibody (green). **B.** Hemocytes expressing *UAS-GFP* with the combined *Hml*^*Δ*^*-* and *He-GAL4* drivers, either alone (*HH*>) or combined with *ird1-*RNAi, (*HH*>*ird1*^*IR*^), and stained with phalloidin only. **C.** Circularity of hemocytes expressing *ird1*^*IR*^ with the *Cg-GAL4* driver (*Cg*>*ird1*^IR^), driver control cells (*+/Cg*) and hemocytes from *ird1*^*Δvps15*^ heterozygotes (*ird1*^*-/+*^) or *ird1*^*Δvps15*^/*ird1*^*5*^ transheterozygous null (*ird1*^*-/-*^) larvae. Blood cells were visualized as in A and their shape assessed in microscopic images using the ImageJ software, with 1.0 indicating perfect circularity. Box-plots indicate medians, quartiles and outliers with the number of analyzed blood cells for each genotype depicted within whiskers. Values for significant differences from pairwise comparisons using Kruskal-Wallis ANOVA are indicated in C. Blood cell images in A and B are maximum intensity projections of *z*-stacks generated by confocal microscopy.
(PDF)

S1 Table Mobilization index (MI) for the deficiencies tested in the screen. (PDF)

S1 Text Detailed characterization of *Tl*^*10b*^ suppressor regions. (PDF)
