# Supplementary material for: Genetic Screen in Drosophila Larvae Links ird1 Function to Toll Signaling in the Fat Body and Hemocyte Motility
Source: PLoS One. 2016 Jul 28;11(7):e0159473. doi: 10.1371/journal.pone.0159473 (PMC4965076; doi:10.1371/journal.pone.0159473)
Supplement: S1 Fig — A. Mapping of suppressive region 1, using Exelixis (Exel) and DrosDel (ED) deletions, to the region highlighted in light grey. Horizontal lines indicate the lengths (in kb) and relative positions of strong suppressive (red) and weak or non-suppressive deficiencies (black). Green and red dashed lines indicate the mobilization indices calculated for wild-type (+) and Tl10b mutant control crosses, respectively. B. Average mobilization indices, calculated from all scored heterozygous Rab23 mutant larvae and the corresponding controls. The total numbers of analyzed individuals and the standard deviations are indicated. Values above the bars represent significance levels from pairwise comparisons to the average mobilization index of Tl10b mutant control crosses, as estimated using Kruskal-Wallis ANOVA. C. Mapping of suppressor region 2, presented as in panel A. D. Average mobilization indices, calculated from all scored larvae of selected mutants, deficiencies and controls from suppressive region 2, presented as in panel B. (PDF) [file pone.0159473.s001.pdf]

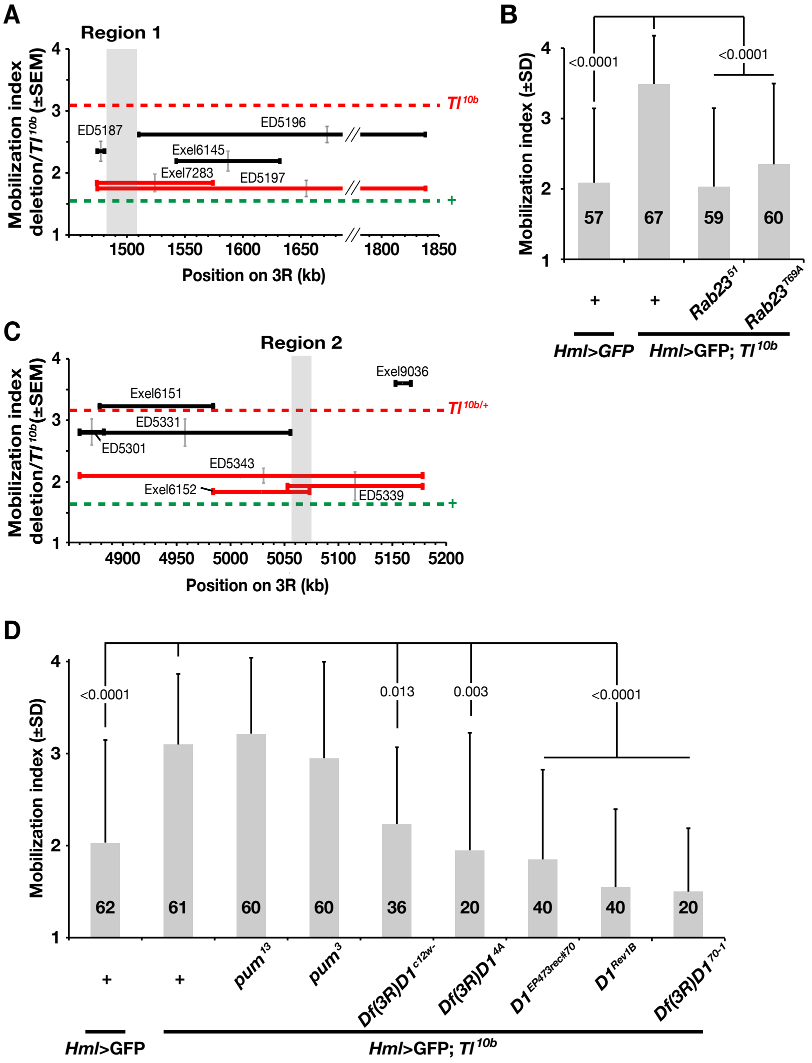

S1 Fig. Deletions over the strong suppressor regions 1 and 2, and mutant alleles of *Rab23*, suppress *Tl*^{10b}-induced loss of hemocyte bands. A. Mapping of suppressive region 1, using Exelixis (Exel) and DrosDel (ED) deletions, to the region highlighted in light grey. Horizontal lines indicate the lengths (in kb) and relative positions of strong suppressive (red) and weak or non-suppressive deficiencies (black). Green and red dashed lines indicate the mobilization indices calculated for wild-type (+) and *Tl*^{10b} mutant control crosses, respectively. **B.** Average mobilization indices, calculated from all scored heterozygous *Rab23* mutant larvae and the corresponding controls. The total numbers of analyzed individuals and the standard deviations are indicated. Values above the bars represent significance levels from pairwise comparisons to the average mobilization index of *Tl*^{10b} mutant control crosses, as estimated using Kruskal-Wallis ANOVA. **C.** Mapping of suppressor region 2, presented as in panel A. **D.** Average mobilization indices, calculated from all scored larvae of selected mutants, deficiencies and controls from suppressive region 2, presented as in panel B.
