# Supplementary material for: Genetic Screen in Drosophila Larvae Links ird1 Function to Toll Signaling in the Fat Body and Hemocyte Motility
Source: PLoS One. 2016 Jul 28;11(7):e0159473. doi: 10.1371/journal.pone.0159473 (PMC4965076; doi:10.1371/journal.pone.0159473)
Supplement: S2 Fig — A. Sessile hemocytes, visualized with HmlΔ-Gal4-driven UAS-GFP, in wild-type control (+) or mutant larvae, carrying either Tl10b alone or combined with Df6332 or hdc loss-of-function alleles (Fus6 or Fus6-50). The same driver is used to express different UAS-hdc RNAi (hdcIR) constructs (39876GD, 39877GD, 45069GD and 104322KK) in hemocytes of Tl10b larvae, or a full-length hdc construct (hdcFL) in hdcFus6-50/ Tl10b heterozygote genetic background. The larvae are oriented with the anterior end up. The bar below each image represents the average mobilization index calculated from the indicated number of larvae of the same genotype. P values were calculated from pairwise comparisons with Tl10b mutant control cross, using Kruskal-Wallis ANOVA test. B. Suppression of hdc expression in hemocytes by different UAS-hdc-RNAi constructs, compared to the HmlΔ-Gal4 driver alone, assayed in hemolymph by quantitative PCR. (PDF) [file pone.0159473.s002.pdf]

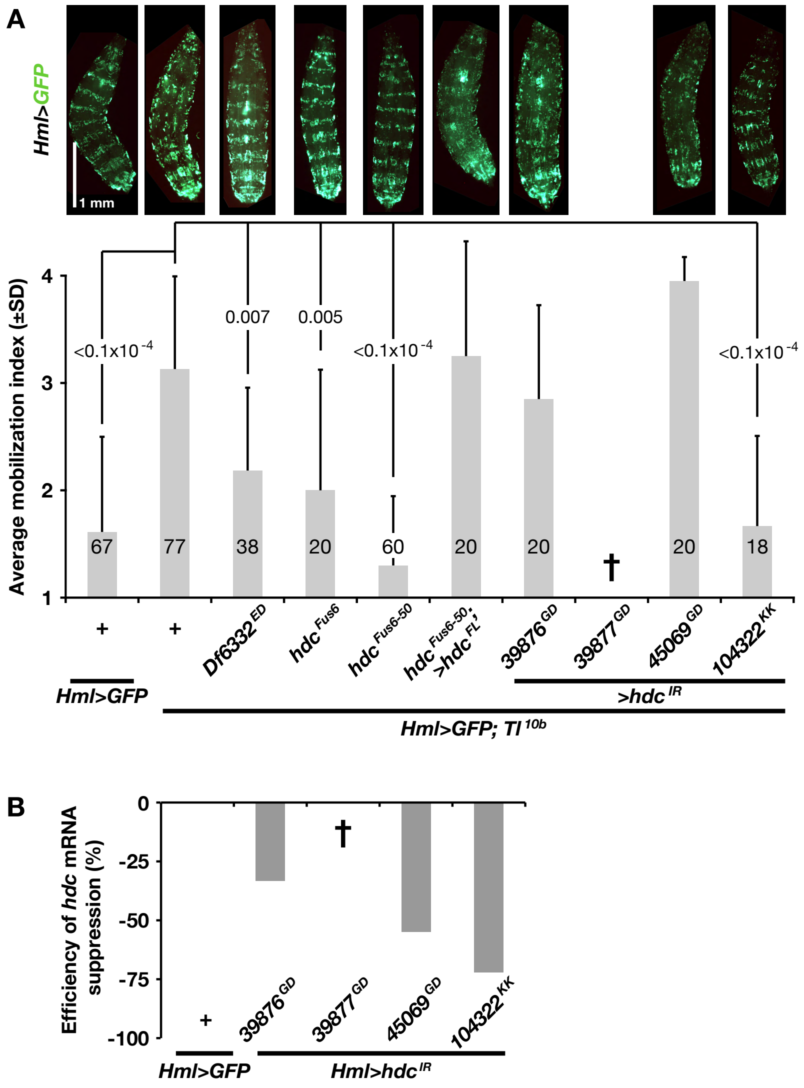

S2 Fig. Loss-of-function alleles and hemocyte-specific RNAi attribute the suppressor effect of *Df6332* to *hdc*. A. Sessile hemocytes, visualized with *Hml*$^{\Delta}$*-Gal4*-driven *UAS-GFP*, in wild-type control (+) or mutant larvae, carrying either *Tl*^{10b} alone or combined with *Df6332* or *hdc* loss-of-function alleles (*Fus6* or *Fus6-50*). The same driver is used to express different UAS-*hdc* RNAi (*hdc*^{IR}) constructs (39876GD, 39877GD, 45069GD and 104322KK) in hemocytes of *Tl*^{10b} larvae, or a full-length *hdc* construct (*hdc*^{FL}) in *hdc*$^{Fus6\text{-}50}$/ *Tl*^{10b} heterozygote genetic background. The larvae are oriented with the anterior end up. The bar below each image represents the average mobilization index calculated from the indicated number of larvae of the same genotype. *P* values were calculated from pairwise comparisons with *Tl*^{10b} mutant control cross, using Kruskal-Wallis ANOVA test. **B.** Suppression of *hdc* expression in hemocytes by different UAS-*hdc*-RNAi constructs, compared to the *Hml*$^{\Delta}$*-Gal4* driver alone, assayed in hemolymph by quantitative PCR.
