# Supplementary material for: Genetic Screen in Drosophila Larvae Links ird1 Function to Toll Signaling in the Fat Body and Hemocyte Motility
Source: PLoS One. 2016 Jul 28;11(7):e0159473. doi: 10.1371/journal.pone.0159473 (PMC4965076; doi:10.1371/journal.pone.0159473)
Supplement: S3 Fig — A. Mean fraction of larvae with at least one melanotic nodule in three independent crosses, with 50 inspected larvae per indicated genotype and cross. B. Mean fraction of circulating blood cells that express HmlΔ-Gal4-driven UAS-GFP fluorescence in hemocyte smears from larvae that carry the driver, alone or together with UAS-ird1IR (>ird1IR) in wild-type (+) or Tl10b genetic background. The number of analyzed images is indicated within the bars and the significance level as estimated by Mann-Whitney U exact test (two-tailed) above. C. The plasmatocyte-specific reporter eater-DsRed visualizes the pattern of sessile cells in third instar larvae heterozygous for the Tl10b allele (Tl10b/+), alone or in combination with either the heterozygous ird1Δvps15 allele (ird1Δ/+) or HmlΔ-Gal4 (Hml>) driven ird1IR, compared to the wild-type control (+). The UAS-GFP construct was present on the same chromosome as the HmlΔ-Gal4 driver, but the green channel is not shown here. The white frame indicates the segmental area used to quantify the number of sessile cells in Fig 3. D. The number of hemocytes in hemolymph from ird1 null (ird15/ird1Δvps15) larvae compared to heterozygous controls (+/ird1Δvps15) and larvae carrying Cg-GAL4 (Cg>) or HmlΔ-Gal4 (Hml>) combined with either He-GAL4 (Hml>He>) or FB-GAL4 (Hml>FB>) drivers, alone or in combination with ird1IR. Box-plots show medians, quartiles and outliers of hemocyte numbers from 10 larvae per genotype. Significance values for differences as estimated by pairwise comparisons using Kruskal-Wallis ANOVA test. Non-significant differences are not indicated. (PDF) [file pone.0159473.s003.pdf]

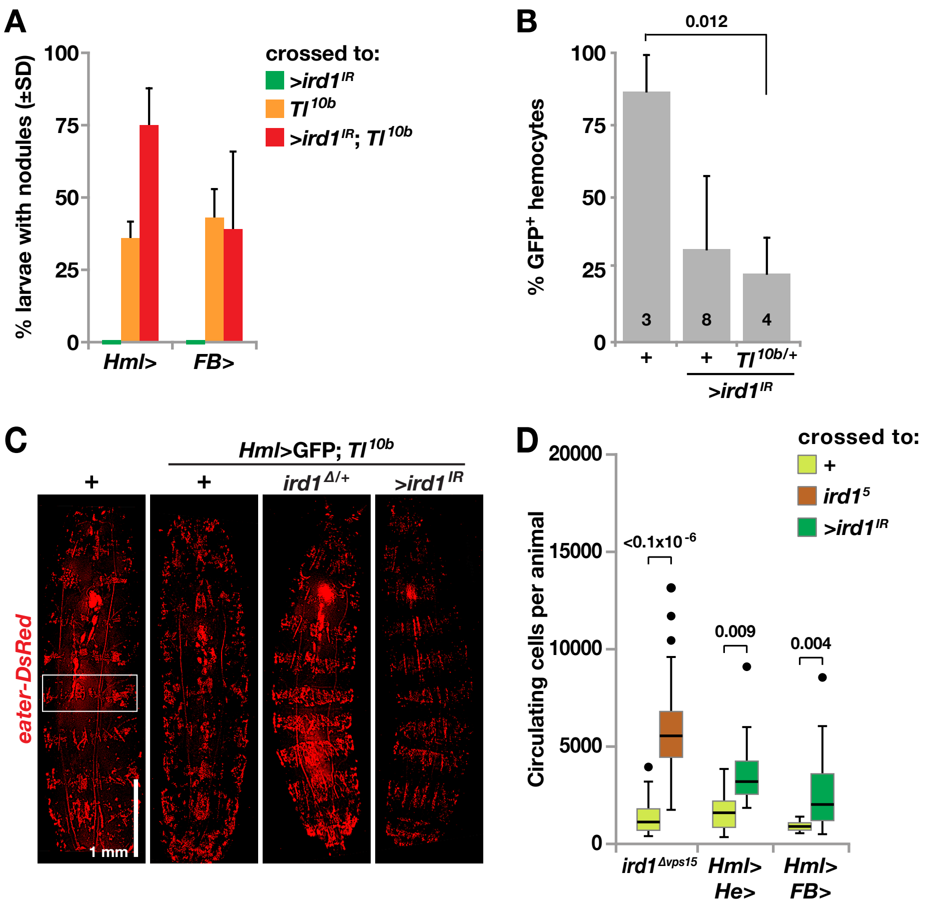

S3 Fig. Hemocyte phenotypes of *ird1* mutants and in *ird1*-suppressed larvae, with or without the *Tl*^{10b} allele. A. Mean fraction of larvae with at least one melanotic nodule in three independent crosses, with 50 inspected larvae per indicated genotype and cross. **B.** Mean fraction of circulating blood cells that express *Hml*$^{\Delta}$*-Gal4*-driven *UAS-GFP* fluorescence in hemocyte smears from larvae that carry the driver, alone or together with *UAS-ird1*^{IR} (*>ird1*^{IR}) in wild-type (+) or *Tl*^{10b} genetic background. The number of analyzed images is indicated within the bars and the significance level as estimated by Mann-Whitney U exact test (two-tailed) above. **C.** The plasmatocyte-specific reporter *eater-DsRed* visualizes the pattern of sessile cells in third instar larvae heterozygous for the *Tl*^{10b} allele (*Tl*$^{10b/+}$), alone or in combination with either the heterozygous *ird1*$^{\Delta vps15}$ allele (*ird1*$^{\Delta/+}$) or *Hml*$^{\Delta}$*-Gal4* (*Hml>*) driven *ird1*^{IR}, compared to the wild-type control (+). The *UAS-GFP* construct was present on the same chromosome as the *Hml*$^{\Delta}$*-Gal4* driver, but the green channel is not shown here. The white frame indicates the segmental area used to quantify the number of sessile cells in Fig 3. **D.** The number of hemocytes in hemolymph from *ird1* null (*ird1*^{5}/*ird1*$^{\Delta vps15}$) larvae compared to heterozygous controls (+/*ird1*$^{\Delta vps15}$) and larvae carrying *Cg-GAL4* (Cg>) or *Hml*$^{\Delta}$*-Gal4* (*Hml>*) combined with either *He-GAL4* (*Hml>He>)* or *FB-GAL4* (*Hml>FB>*) drivers, alone or in combination with *ird1*^{IR}. Box-plots show medians, quartiles and outliers of hemocyte numbers from 10 larvae per genotype. Significance values for differences as estimated by pairwise comparisons using Kruskal-Wallis ANOVA test. Non-significant differences are not indicated.
