# Supplementary material for: Genetic Screen in Drosophila Larvae Links ird1 Function to Toll Signaling in the Fat Body and Hemocyte Motility
Source: PLoS One. 2016 Jul 28;11(7):e0159473. doi: 10.1371/journal.pone.0159473 (PMC4965076; doi:10.1371/journal.pone.0159473)
Supplement: S4 Fig — A. Fraction of ird1 null (ird1-/-) or ird1 RNAi (>ird1IR) larvae showing at least one blood cell accumulation visualized by the msn-Cherry lamellocyte reporter. The UAS-ird1IR (>ird1IR) construct was expressed with hemocyte (Hml>), fat body (FB> and ppl>), fat body+hemocyte (Cg>), or ubiquitous (da>) drivers. The number of analyzed individuals of each genotype is indicated. B-C. Hemocytes from wild-type (+), Tl10b heterozygous (Tl10b/+), or ird1 functional null (ird1-/-) larvae, carrying the eater-DsRed plasmatocyte reporter, stained with Hoechst nuclear dye (blue) and (B) plasmatocyte- or (C) lamellocyte-specific antibodies (green). Arrowheads and arrows in C indicate lamellocytes that have retained or lost eater-DsRed expression, respectively. D. Expression of the msn-mCherry lamellocyte marker in control (+) and ird1-/- larvae, before and after starvation. Arrowheads indicate lamellocyte accumulations. The msn-mCherry marker was also strongly expressed ectopically in parts of the larval musculature. (PDF) [file pone.0159473.s004.pdf]

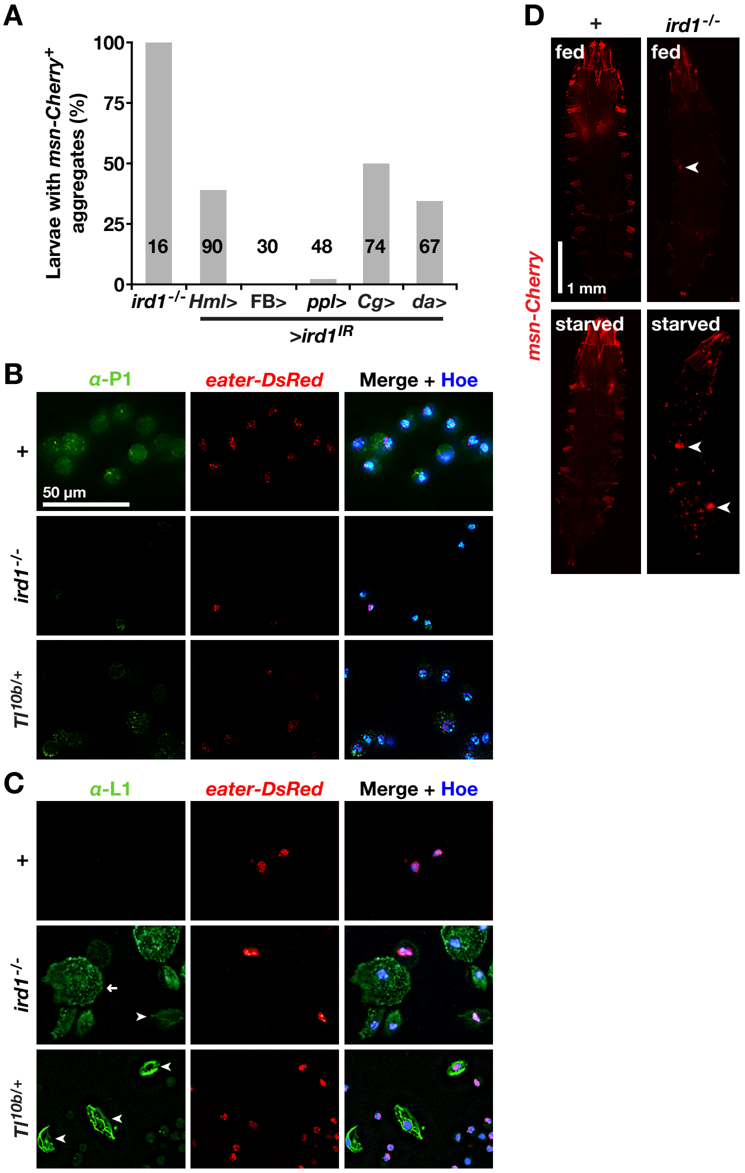

S4 Fig. *ird1* hemocyte phenotypes as seen with *msn-Cherry*, *eater-DsRed*, and antibody markers. A. Fraction of *ird1* null (*ird1*$^{-/-}$) or *ird1* RNAi (>*ird1*^{IR}) larvae showing at least one blood cell accumulation visualized by the *msn-Cherry* lamellocyte reporter. The *UAS-ird1*^{IR} (>*ird1*^{IR}) construct was expressed with hemocyte (*Hml*>), fat body (*FB*> and *ppl*>), fat body+hemocyte (*Cg*>), or ubiquitous (*da*>) drivers. The number of analyzed individuals of each genotype is indicated. **B-C.** Hemocytes from wild-type (+), *Tl*^{10b} heterozygous (*Tl*$^{10b/+}$), or *ird1* functional null (*ird1*$^{-/-}$) larvae, carrying the *eater-DsRed* plasmatocyte reporter, stained with Hoechst nuclear dye (blue) and (B) plasmatocyte- or (C) lamellocyte-specific antibodies (green). Arrowheads and arrows in C indicate lamellocytes that have retained or lost *eater-DsRed* expression, respectively. **D.** Expression of the *msn-mCherry* lamellocyte marker in control (+) and *ird1*$^{-/-}$ larvae, before and after starvation. Arrowheads indicate lamellocyte accumulations. The *msn-mCherry* marker was also strongly expressed ectopically in parts of the larval musculature.
