# Supplementary material for: Genetic Screen in Drosophila Larvae Links ird1 Function to Toll Signaling in the Fat Body and Hemocyte Motility
Source: PLoS One. 2016 Jul 28;11(7):e0159473. doi: 10.1371/journal.pone.0159473 (PMC4965076; doi:10.1371/journal.pone.0159473)
Supplement: S5 Fig — A. Spontaneous melanotic nodule formation (arrowhead) in an ird15/ird1Δvps15 (ird1-/-) transheterozygous larva. B. Posterior parts of wild-type control (+) and ird1-/- larvae, 4 h after injection with an E. coli suspension (upper panels), and filter paper incubated together with the infected animals (lower panel). C and D. Toll pathway activation, as detected by the Drs-GFP reporter, in larval fat bodies (C) or extracted hemocytes (D) of ird1Δvps15 heterozygotes (ird1+/-), or in larvae expressing UAS-Tl10b (>Tl10b) or UAS-ird1-RNAi (>ird1IR) in fat body alone by the FB-Gal4 (FB>) driver, or in fat body and hemocytes by the Cg-GAL4 (Cg>) driver. Drs-GFP expression is also visible in sessile (C) and circulating (D) blood cell populations of larvae expressing UAS-Tl10b in hemocytes only by the HmlΔ-Gal4 (Hml>) driver. (PDF) [file pone.0159473.s005.pdf]

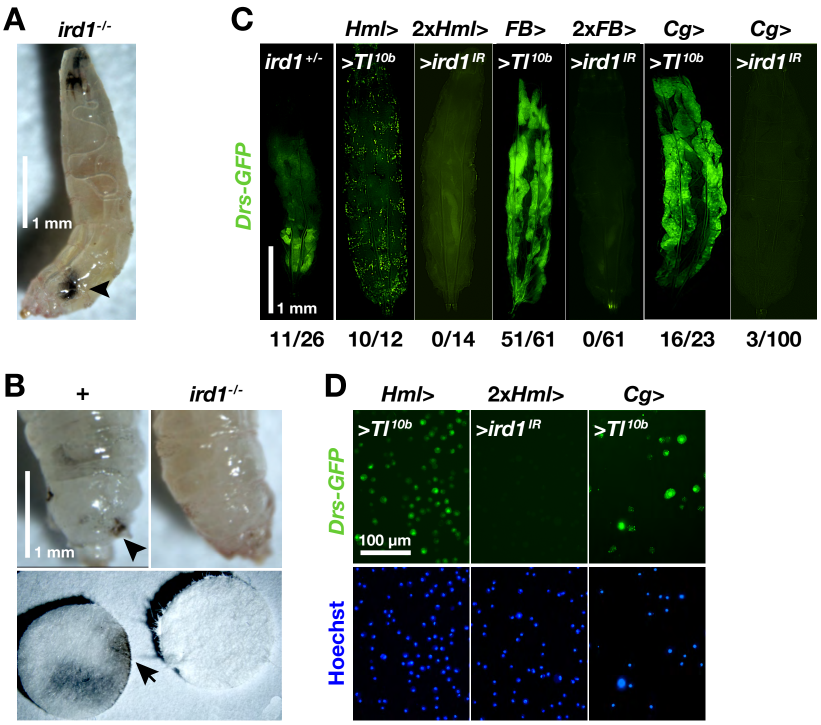

S5 Fig. *ird1* null mutant larvae have melanization defects. *ird1* heterozygotes but not *ird1*^{IR} animals show Toll pathway activation in the fat body. A. Spontaneous melanotic nodule formation (arrowhead) in an *ird1*^{5}/*ird1*$^{\Delta vps15}$ (*ird1*$^{-/-}$) transheterozygous larva. **B.** Posterior parts of wild-type control (+) and *ird1*$^{-/-}$ larvae, 4 h after injection with an *E. coli* suspension (upper panels), and filter paper incubated together with the infected animals (lower panel). **C and D.** Toll pathway activation, as detected by the *Drs-GFP* reporter, in larval fat bodies (C) or extracted hemocytes (D) of *ird1*$^{\Delta vps15}$ heterozygotes (*ird1*$^{+/-}$), or in larvae expressing *UAS-Tl*^{10b} (>*Tl*^{10b}) or *UAS-ird1*-RNAi (>*ird1*^{IR}) in fat body alone by the *FB-Gal4* (*FB*>) driver, or in fat body and hemocytes by the *Cg-GAL4* (*Cg*>) driver. *Drs-GFP* expression is also visible in sessile (C) and circulating (D) blood cell populations of larvae expressing *UAS-Tl*^{10b} in hemocytes only by the *Hml*$^{\Delta}$*-Gal4* (*Hml*>) driver.
