# Supplementary material for: Genetic Screen in Drosophila Larvae Links ird1 Function to Toll Signaling in the Fat Body and Hemocyte Motility
Source: PLoS One. 2016 Jul 28;11(7):e0159473. doi: 10.1371/journal.pone.0159473 (PMC4965076; doi:10.1371/journal.pone.0159473)
Supplement: S6 Fig — A. Total number of Atg8a-GFP and/or Atg8a-mCherry-labeled vesicles per cell. B. The fraction of vesicles in each cell that was labeled with Atg8a-mCherry only. Each dot represents the count from a single cell and the black bars indicate median values. P values for pairwise comparisons using Kruskal-Wallis ANOVA test are shown above. (PDF) [file pone.0159473.s006.pdf]

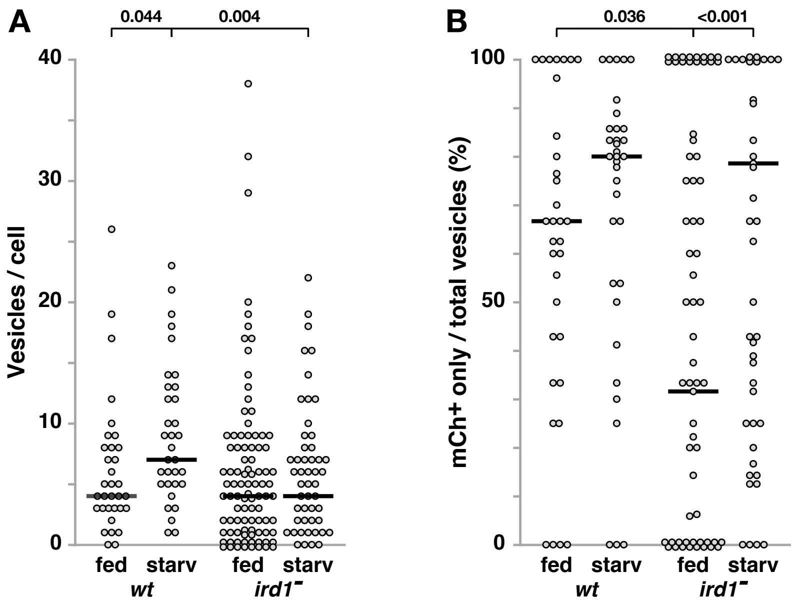

S6 Fig. *ird1* knockdown blocks the starvation-induced increase in autophagosome and autolysosome numbers in hemocytes and reduces the fraction of hemocyte autolysosomes in fed larvae A. Total number of *Atg8a*-GFP and/or *Atg8a*-mCherry-labeled vesicles per cell. **B.** The fraction of vesicles in each cell that was labeled with *Atg8a*-mCherry only. Each dot represents the count from a single cell and the black bars indicate median values. *P* values for pairwise comparisons using Kruskal-Wallis ANOVA test are shown above.
