# Supplementary material for: Genetic Screen in Drosophila Larvae Links ird1 Function to Toll Signaling in the Fat Body and Hemocyte Motility
Source: PLoS One. 2016 Jul 28;11(7):e0159473. doi: 10.1371/journal.pone.0159473 (PMC4965076; doi:10.1371/journal.pone.0159473)
Supplement: S7 Fig — A. Average mobilization index and B percentage of animals expressing UAS-GFP with blood cell specific HmlΔ-Gal4 (Hml>) driver with at least one melanotic nodule among wild-type control, or Tl10b larvae with or without the indicated loss-of-function alleles. Three independent experiments were performed for each genotype, 20 larvae were graded and 50 were inspected for nodules in each. Significant difference (***, p<0.0001; **, p<0.001) compared to the Tl10b mutant control, as estimated by pairwise comparisons using Kruskal-Wallis ANOVA test. Non-significant differences are not indicated. (PDF) [file pone.0159473.s007.pdf]

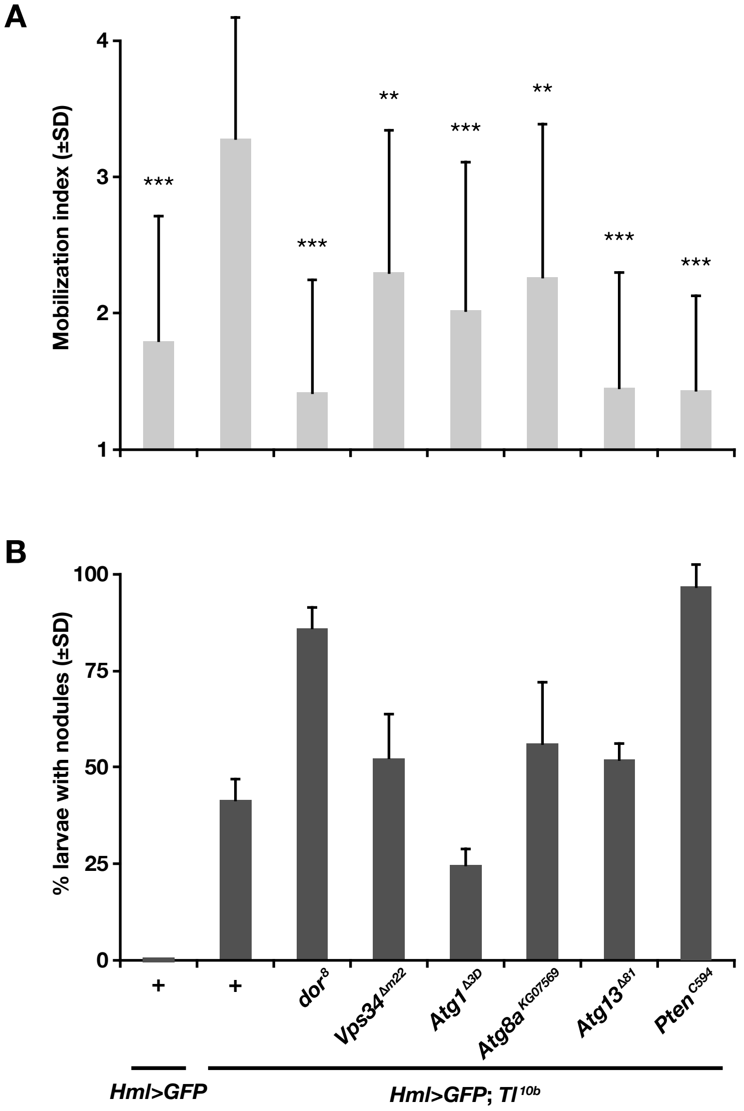

S7 Fig. Modification of *Tl¹⁰ᵇ* phenotype by mutants of vesicle transport genes. A. Average mobilization index and **B** percentage of animals expressing *UAS-GFP* with blood cell specific *Hml^{Δ}-Gal4* (*Hml>*) driver with at least one melanotic nodule among wild-type control, or *Tl^{10b}* larvae with or without the indicated loss-of-function alleles. Three independent experiments were performed for each genotype, 20 larvae were graded and 50 were inspected for nodules in each. Significant difference (***, *p*<0.0001; **, *p*<0.001) compared to the *Tl^{10b}* mutant control, as estimated by pairwise comparisons using Kruskal-Wallis ANOVA test. Non-significant differences are not indicated.
