# Supplementary material for: Genetic Screen in Drosophila Larvae Links ird1 Function to Toll Signaling in the Fat Body and Hemocyte Motility
Source: PLoS One. 2016 Jul 28;11(7):e0159473. doi: 10.1371/journal.pone.0159473 (PMC4965076; doi:10.1371/journal.pone.0159473)
Supplement: S8 Fig — A. Two examples of hemocytes expressing UAS-Vps34KD with Cg-GAL4 (Cg>Vps34KD) spread on glass slides and stained with DAPI nuclear dye (blue), AlexaFluor-conjugated phalloidin (red) and FITC-conjugated α-tubulin antibody (green). B. Hemocytes expressing UAS-GFP with the combined HmlΔ- and He-GAL4 drivers, either alone (HH>) or combined with ird1-RNAi, (HH>ird1IR), and stained with phalloidin only. C. Circularity of hemocytes expressing ird1IR with the Cg-GAL4 driver (Cg>ird1IR), driver control cells (+/Cg) and hemocytes from ird1Δvps15 heterozygotes (ird1-/+) or ird1Δvps15/ird15 transheterozygous null (ird1-/-) larvae. Blood cells were visualized as in A and their shape assessed in microscopic images using the ImageJ software, with 1.0 indicating perfect circularity. Box-plots indicate medians, quartiles and outliers with the number of analyzed blood cells for each genotype depicted within whiskers. Values for significant differences from pairwise comparisons using Kruskal-Wallis ANOVA are indicated in C. Blood cell images in A and B are maximum intensity projections of z-stacks generated by confocal microscopy. (PDF) [file pone.0159473.s008.pdf]

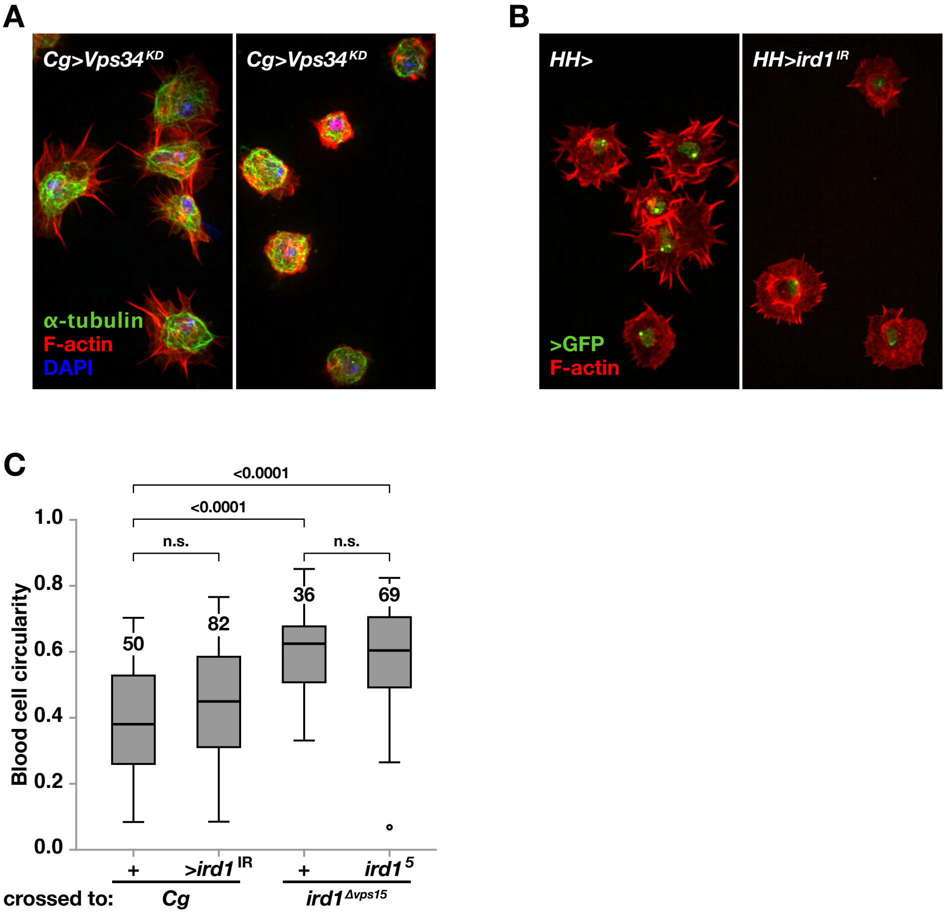

S8 Fig. Effects of *Vps34* and *ird1* suppression on hemocyte morphology. A. Two examples of hemocytes expressing *UAS-Vps34*^{KD} with *Cg-GAL4* (*Cg>Vps34*^{KD}) spread on glass slides and stained with DAPI nuclear dye (blue), AlexaFluor-conjugated phalloidin (red) and FITC-conjugated α-tubulin antibody (green). **B.** Hemocytes expressing *UAS-GFP* with the combined *Hml*$^{\Delta}$- and *He-GAL4* drivers, either alone (*HH>*) or combined with *ird1*-RNAi, (*HH>ird1*^{IR}), and stained with phalloidin only. **C.** Circularity of hemocytes expressing *ird1*^{IR} with the *Cg-GAL4* driver (*Cg>ird1*^{IR}), driver control cells (+/*Cg*) and hemocytes from *ird1*$^{\Delta vps15}$ heterozygotes (*ird1*$^{-/+}$) or *ird1*$^{\Delta vps15}$/*ird1*^{5} transheterozygous null (*ird1*$^{-/-}$) larvae. Blood cells were visualized as in A and their shape assessed in microscopic images using the ImageJ software, with 1.0 indicating perfect circularity. Box-plots indicate medians, quartiles and outliers with the number of analyzed blood cells for each genotype depicted within whiskers. Values for significant differences from pairwise comparisons using Kruskal-Wallis ANOVA are indicated in C. Blood cell images in A and B are maximum intensity projections of *z*-stacks generated by confocal microscopy.
