# Supplementary material for: Genetic Screen in Drosophila Larvae Links ird1 Function to Toll Signaling in the Fat Body and Hemocyte Motility
Source: PLoS One. 2016 Jul 28;11(7):e0159473. doi: 10.1371/journal.pone.0159473 (PMC4965076; doi:10.1371/journal.pone.0159473)
Supplement: S1 Table — (PDF) [file pone.0159473.s009.pdf]

S1 Table. Mobilization index (MI) for the deficiencies tested in the screen

	Deficiency ID	N(crosses)	N(larvae)	MI	SEM	Genes
1	Df(3R)ED5020	6	41	2.54	0.30	*tube*
2	Df(3R)ED5142	4	14	2.77	0.38	
3	Df(3R)ED5066	1	10	3.70		
4	Df(3R)ED5138	4	29	3.14	0.16	
5	Df(3R)ED5147	1	15	2.67		
6	Df(3R)ED5156	2	27	2.92	0.11	
7	Df(3R)ED10257	1	17	**2.41**	0.19	
8	Df(3R)ED5177	4	24	2.83	0.43	
9	Df(3R)Exel7283	2	32	**1.86**	0.34	*Rab23*
10	Df(3R)ED5187	3	47	**2.47**	0.36	
11	Df(3R)ED5197	7	70	**1.75**	0.34	*Rab23*
12	Df(3R)ED5196	4	39	2.62	0.18	*Rab23*
13	Df(3R)Exel6145	2	32	**2.18**	0.05	
14	Df(3R)Exel7284	2	36	**1.77**	0.35	
15	Df(3R)ED7665	2	21	**2.47**	0.18	
16	Df(3R)ED5221	4	35	2.63	0.28	
17	Df(3R)ED5223	5	39	**2.31**	0.25	
18	Df(3R)ED5220	4	38	2.84	0.10	
19	Df(3R)ED5230	5	42	2.69	0.14	
20	Df(3R)ED5301	2	32	2.81	0.31	
21	Df(3R)ED5331	2	32	2.80	0.34	
22	Df(3R)ED5343	4	40	**2.10**	0.20	*pum/D1/ird1*
23	Df(3R)Exel6151	2	36	3.19	0.34	
24	Df(3R)Exel6152	2	32	**1.84**	0.09	*pum/D1/ird1*
25	Df(3R)ED5339	2	34	**1.85**	0.07	*pum/D1/ird1*
26	Df(3R)Exel9036	2	36	3.61	0.01	
27	Df(3R)ED5428	1	16	**2.30**		
28	Df(3R)ED5438	2	15	**2.47**	0.08	
29	Df(3R)ED5454	3	24	2.67	0.35	
30	Df(3R)ED5474	1	15	3.27		
31	Df(3R)ED5495			**lethal**		
32	Df(3R)ED5506	4	28	**2.36**	0.16	
33	Df(3R)ED5518	1	15	3.00		
34	Df(3R)ED5516	1	16	3.06		
35	Df(3R)ED5559			**lethal**		
36	Df(3R)ED5554	3	16	2.50	0.15	
37	Df(3R)ED5573			**lethal**		
38	Df(3R)ED5591	4	21	2.62	0.28	
39	Df(3R)ED5610	2	33	2.79	0.32	
40	Df(3R)Exel6166	2	34	3.38	0.21	
41	Df(3R)Exel7318	2	35	**2.21**	0.21	
42	Df(3R)ED5608	4	60	**1.78**	0.19	
43	Df(3R)ED5612	5	55	**2.31**	0.18	
44	Df(3R)ED5613	3	26	2.69	0.28	
45	Df(3R)ED5623			**lethal**		
46	Df(3R)ED5644	1	17	2.90		
47	Df(3R)Exel8160	1	18	2.72		
48	Df(3R)ED5664	2	20	3.05	0.22	
49	Df(3R)Exel6275	1	10	**2.30**		
50	Df(3R)ED10556	1	16	3.05		

(continued)

S1 Table (continued)

	Deficiency ID	N(crosses)	N(larvae)	MI	SEM	Genes
51	Df(3R)Exel6172	1	18	**2.28**		
52	Df(3R)Exel6173	1	17	3.53		
53	Df(3R)ED5688	1	16	3.56		
54	Df(3R)ED5705			**lethal**		
55	Df(3R)ED10639	1	16	2.56		
56	Df(3R)ED10642	3	45	2.69	0.52	
57	Df(3R)ED5780	3	33	**1.76**	0.30	
58	Df(3R)Exel6176	1	15	**1.73**		
59	Df(3R)ED5781	3	32	**2.19**	0.37	
60	Df(3R)ED5794			**lethal**		
61	Df(3R)ED5815	2	18	2.61	0.08	
62	Df(3R)ED2	1	11	3.27		
63	Df(3R)ED5911	3	14	2.57	0.29	
64	Df(3R)ED6025	4	34	2.74	0.15	
65	Df(3R)ED10820	1	17	2.53		
66	Df(3R)ED10845	1	11	2.91		
67	Df(3R)ED6052	1	16	3.39		
68	Df(3R)ED6058			**lethal**		
69	Df(3R)ED6076	2	20	**2.35**	0.32	
70	Df(3R)ED6093			**lethal**		
71	Df(3R)ED6096	2	21	2.57	0.31	
72	Df(3R)ED6103	3	22	2.68	0.25	
73	Df(3R)ED6105	3	46	**2.37**	0.24	
74	Df(3R)ED10894	1	16	2.94		
75	Df(3R)ED6187			**lethal**		
76	Df(3R)ED6220	2	31	**2.35**	0.65	
77	Df(3R)ED10951	1	15	2.80		
78	Df(3R)Exel6204	2	34	**1.71**	0.18	
79	Df(3R)ED6232	3	39	**1.67**	0.31	
80	Df(3R)ED6235	1	18	**1.33**		
81	Df(3R)ED6255	2	19	2.79	0.49	*Tl/spz/pll*
82	Df(3R)ED6265	4	28	2.93	0.19	*pll*
83	Df(3R)ED10970	1	17	2.82		
84	Df(3R)ED6310	2	11	2.64	0.02	
85	Df(3R)ED6316	3	23	2.87	0.25	
86	Df(3R)ED6332	3	38	**2.17**	0.29	*hdc*
87	Df(3R)ED6346	1	12	2.92		
88	Df(3R)ED6361	1	16	3.00		

Strong suppressor
Weak suppressor
Lethal
