# Supplementary material for: Genetic Screen in Drosophila Larvae Links ird1 Function to Toll Signaling in the Fat Body and Hemocyte Motility
Source: PLoS One. 2016 Jul 28;11(7):e0159473. doi: 10.1371/journal.pone.0159473 (PMC4965076; doi:10.1371/journal.pone.0159473)
Supplement: S1 Text — (PDF) [file pone.0159473.s010.pdf]

S1 Text. Detailed characterization of *Tl^10b* suppressor regions.

Strong suppressor region 1: alleles of *Rab23* suppress *Tl^10b* induced loss of sessile blood cell bands

With the help of additional deficiencies, region one was mapped down from originally including almost 360 kilobase pairs (kb) to just 30 kb. Its suppressive nature could be independently confirmed by a deletion (*Df7283^EX*) originally created for the Exelixis deficiency kit (Fig. S1A). Due to time constrains, only one out of seven genes included in region 1 was tested (Table 1). Coding for a member of the small GTPase protein family, *Rab23* is mostly known for being a negative regulator of Hedgehog signaling in mouse development [1,2]. We obtained the alleles *Rab23^T69A* and *Rab23^51*, which were previously described as strong-loss-of function or null alleles of *Rab23* [3]. When crossed independently to *Hml^Δ-Tl^10b*, both alleles produced larvae with significantly lower MI compared to the mutant control crosses (Fig. S1B). However, *Df5196^ED* (a deletion that with an MI of 2.62 fell just outside our category for weak suppressors) also covers the transcription start side of *Rab23*. Therefore, it cannot be excluded at this point that the gene responsible for the strong effect of suppressor region 1 is not *Rab23* itself, but one or more of the other genes situated in its proximity.

Strong suppressor region 2: individual tested genes

Using additional deficiencies, we could map down the strong suppressor region 2 from 318 to 18 kb including only five genes (Fig. S1 C and Table 1). We tested the suppression of the Tl^{10b} phenotype in mutants for three of the putative suppressors, the translational repressor gene *pumilio* (*pum*), the heterochromatin protein gene *D1*, and the PI3K subunit gene *ird1*. We made no effort to investigate the remaining two genes, CG8420 and the non-protein coding gene CR4519, for which neither mutant stocks nor information about molecular function were available. For *pumilio* we tested two homozygous lethal alleles, *pum^3* and *pum^13*, the latter being a known loss-of-function mutation [4], but neither of them replicated the suppressive effect of the smallest deficiency of this region (*Df6152^EX*), when crossed to *Hml^Δ-Tl^10b*, with mobilization indices similar to those of *Tl^10b* control larvae (Fig. S1D). In contrast, our results with the D1 gene were inconclusive. A hypomorph P element insertion allele of the *D1* gene, *D1^EP473*, as well as two deletion mutants generated by excision of this element, *Df(3R)D1^c12w-* and *Df(3R)D1^4A* [5], produced larvae with significantly lower mobilization indices compared to the mutant control (Fig. S1D) However wild-type D1 revertant stocks (*D1^Rev1B*, *Df(3R)D1^70-1*), created by precise excision of the the *P*-element in the original P-insertion hypomorph stocks [5], gave similar or even stronger suppression of our phenotype. We conclude that the suppressor activity of these *D1* stocks must reside in the genetic background and therefore does not depend on the *D1* gene itself. Finally, two *ird1* mutants, *ird1^5* and *ird1^Δvps15*, turned out to be true suppressors of the *Tl^10b* phenotype, as described in the main text (Fig. 2).

A weak suppressor region: *hdc* gene dosage suppresses loss of sessile bands, validating the screening approach

With a mobilization index of 2.17, the deficiency stock *Df6332^ED* falls just outside our set border for strong suppressors. It is comparatively small, including only four genes (see Fig. 1 C and Table 1). Among these genes, *headcase* (*hdc*) was by far the largest and the only gene for which mutant fly strains were available. Named for its role in adult head formation, it was the first gene reported to be specifically expressed in all imaginal cells of the *Drosophila* larva [6]. As a proof of principle, we obtained two *hdc* mutant strains (*Fus-6* and *Fus-6^50*), previously described as homozygous lethal and causing additional branching in the embryonic tracheal network [7,8]. Larval offspring produced by crossing these alleles independently to *Hml^Δ-Tl^10b* scored on average with significantly lower mobilization indices compared to the mutant con-

trol cross (Fig. S2A). This suppressive effect was entirely lost when we expressed a full-length *hdc* genomic construct specifically in blood cells of larvae heterozygous for $hdc^{Fus\text{-}6\text{-}50}$ and Tl^{10b}. When we tried to replicate the result obtained with the original deletion and the *hdc* mutants by expressing RNAi constructs for *hdc* (hdc^{IR}) [9], only one (104322KK) out of four tested fly stocks suppressed the Tl^{10b} phenotype (Fig. S2A and B). The other three had either no effect (39876GD and 45069GD), or conveyed lethality (39877GD). Besides the fact that 104322KK is the only construct that affected our phenotype, it is also the only one targeting all three *hdc* isoforms [10]. Analysis of mRNA levels by quantitative real-time PCR suggests that it is also the most efficient construct when it comes to silencing *hdc* expression in blood cells (Fig. S2B). Apart from verifying the feasibility of our approach, these findings indicate that *hdc* expression in hematopoietic tissues is required for the responsiveness of sessile blood cells to Toll pathway signaling, and possibly for the general development of the larva. The encapsulation of *L. boulardi* parasitoid wasp eggs was also reduced in Hml^{Δ}-*GAL4* larvae expressing the 104332KK *hdc*-RNAi construct (data not shown).

References

1. Eggenschwiler JT, Bulgakov OV, Qin J, Li T, Anderson KV. Mouse Rab23 regulates Hedgehog signaling from Smoothened to Gli proteins. Dev Biol. 2006;290: 1-12.
2. Nozawa T, Aikawa C, Goda A, Maruyama F, Hamada S, Nakagawa I. The small GTPases Rab9A and Rab23 function at distinct steps in autophagy during Group A Streptococcus infection. Cell Microbiol. 2012;14: 1149-1165.
3. Pataki C, Matusek T, Kurucz É, Andó I, Jenny A, Mihály J. Drosophila Rab23 is involved in the regulation of the number and planar polarization of the adult cuticular hairs. Genetics. 2010;184: 1051-1065.
4. Wharton RP, Sonoda J, Lee T, Patterson M, Murata Y. The Pumilio RNA-binding domain is also a translational regulator. Mol Cell. 1998;1: 863-872.
5. Weiler KS, Chatterjee S. The multi-AT-hook chromosomal protein of Drosophila melanogaster, D1, is dispensable for viability. Genetics. 2009;182: 145-159.
6. Weaver TA, White RA. *headcase*, an imaginal specific gene required for adult morphogenesis in *Drosophila melanogaster*. Development. 1995;121: 4149-4160.
7. Steneberg P, Englund C, Kronhamn J, Weaver TA, Samakovlis C. Translational readthrough in the *hdc* mRNA generates a novel branching inhibitor in the *Drosophila* trachea. Genes Dev. 1998;12: 956-967.
8. Hartenstein V, Jan YN. Studying *Drosophila* embryogenesis with P-*lacZ* enhancer trap lines. Roux's Arch Dev Biol. 1992;201: 194-220.
9. Dietzl G, Chen D, Schnorrer F, Su KC, Barinova Y, Fellner M, et al. A genome-wide transgenic RNAi library for conditional gene inactivation in *Drosophila*. Nature. 2007;448: 151-156.
10. Resende LPF, Boyle M, Tran D, Fellner T, Jones DL. Headcase promotes cell survival and niche maintenance in the *Drosophila* testis. PLoS One. 2013;8: e68026.
